# Stable 2D anti-ferromagnetically coupled fluorenyl radical dendrons†
†Electronic supplementary information (ESI) available: Synthetic procedures and characterization data of all new compounds; details for all physical characterization and theoretical calculations; and additional spectroscopic data. See DOI: 10.1039/c7sc05493a


**DOI:** 10.1039/c7sc05493a

**Published:** 2018-02-28

**Authors:** Jian Wang, Gakhyun Kim, María Eugenia Sandoval-Salinas, Hoa Phan, Tullimilli Y. Gopalakrishna, Xuefeng Lu, David Casanova, Dongho Kim, Jishan Wu

**Affiliations:** a Department of Chemistry , National University of Singapore , 3 Science Drive 3 , 117543 , Singapore . Email: chmwuj@nus.edu.sg; b Spectroscopy Laboratory for Functional π-Electronic Systems and Department of Chemistry , Yonsei University , Seoul 03722 , Korea . Email: dongho@yonsei.ac.kr; c Kimika Fakultatea , Euskal Herriko Unibertsitatea , Donostia International Physics Center , Paseo Manuel de Lardizabal, 4, 20018, Euskadi , Donostia-San Sebastián , Spain . Email: david.casanova@ehu.eus; d Departament de Ciència de Materials i Química Física , Institut de Química Teòrica i Computacional (IQTCUB) , Universitat de, Barcelona , Martí i Franquès 1-11 , Barcelona 08028 , Spain

## Abstract

We report the first class of stable, two-dimensional (2D) anti-ferromagnetically coupled dendritic polyradicaloids. A kinetically blocked fluorenyl radical was used to build up the first and second generation dendrons **FR-G1** and **FR-G2** containing three and seven fluorenyl units, respectively. The neighboring fluorenyl radicals in **FR-G1** and **FR-G2** show moderate anti-ferromagnetic exchange interaction, resulting in a doublet and quartet ground state, respectively, with a small doublet–quartet energy gap. From **FR-G1** to **FR-G2**, the energy gap decreased and the two-photon absorption was enhanced owing to more extended 2D π-conjugation. Both compounds showed multiple redox waves due to their polyradical character.

## Abstract 2

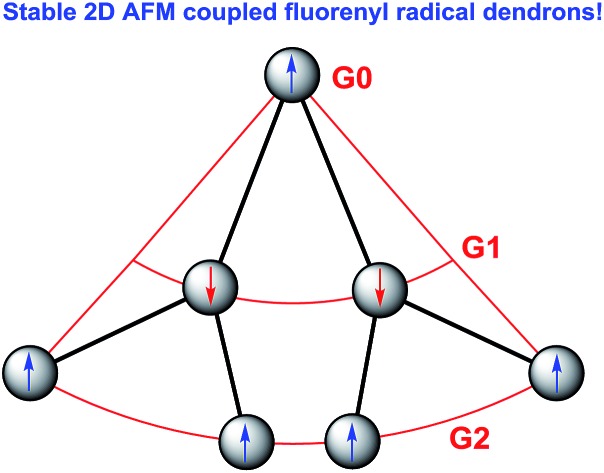
The first class of stable two-dimensional anti-ferromagnetically coupled dendritic polyradicaloids was synthesized, which show polyradical character and unique properties.

## Introduction

**Fig. 1:**
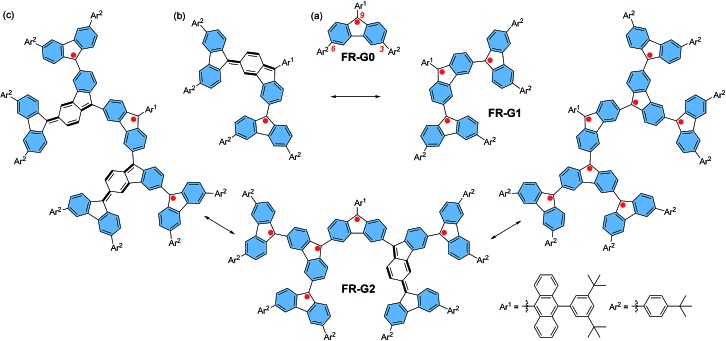
Chemical structures and representative canonical forms of the fluorenyl radical dendrons **FR-G0** (a), **FR-G1** (b) and **FR-G2** (c).

Spin–spin exchange interaction in organic diradicals and polyradicals fundamentally determines their magnetic properties and material applications.^1^ While ferromagnetic (FM) coupling usually leads to a high-spin ground state which is of importance for organic magnets,^2^ anti-ferromagnetic (AFM) exchange interaction helps to enhance electronic conjugation between the radicals and results in remarkable optical, electronic and magnetic properties as recently demonstrated in many open-shell singlet diradicaloids and polyradicaloids.^3^ Topologically, the radicals can be linked in linear, macrocyclic, star-branched, and even dendritic motifs, and the topological symmetry determines the spin multiplicity of the polyradicals. Among them, dendritic polyradicals are particularly interesting as they provide two-dimensional (2D) multiple spin–spin interactions. Rajca's group and Iwamura's group independently developed dendritic polyarylmethyl radicals^4^ and polycarbenes,^5^ respectively, both showing strong FM coupling between the neighbouring radicals with a high-spin ground state. However, these dendritic polyradicals are kinetically unstable and they can only be generated and analysed *in situ* in an inert atmosphere at low temperature. On the other hand, AFM coupled polyradicaloids show much better stability due to the bonding interaction between the radicals and recently, stable linear^6^ and macrocyclic polyradicaloids^7^ have been successfully prepared. However, to the best of our knowledge, stable AFM coupled dendritic polyradicaloids remain unknown. It was previously demonstrated that the fluorenyl radical became stable if the 9-position was kinetically blocked by a bulky anthryl group, such as **FR-G0** in Fig. 1.^6^,^8^ Therefore, we designed the dendritic triradicaloid **FR-G1** and heptaradicaloid **FR-G2** (Fig. 1), in which the 3,6-positions of the inner fluorenyl radical are directly linked to the 9-position of the outer fluorenyl radicals. They can be regarded as the first and second generation fluorenyl radical mono-dendron, respectively. The 9-position of the core fluorenyl unit is kinetically blocked by a bulky 9-(3,5-di-*tert*-butylphenyl)anthryl and the 3,6-positions of the outermost fluorenyls are blocked by 4-*tert*-butylphenyl groups. In addition, the inner fluorenyl unit itself serves as a kinetic blocking group for the outer fluorenyl radicals. Notably, the neighbouring fluorenyl units can form AFM bonding by losing one aromatic sextet ring (the hexagon shaded by blue colour) and generation of a *para*-quinodimethane unit (Fig. 1). As a result, monoradical and triradical/pentaradical resonance forms can also be drawn for **FR-G1** and **FR-G2**, respectively. These 2D AFM coupled and kinetically protected fluorenyl radical dendrons are supposed to be stable and exhibit interesting physical properties. They are also significantly different from the reported polyphenylene dendrimers^9^ or polyphenylacetylene dendrimers,^10^ in which the π-conjugation is usually interrupted at the branch points due to the large distortional angle between the phenyl units or *meta*-phenyl linkage.

## Results and discussion

### Synthesis

**Scheme 1:**
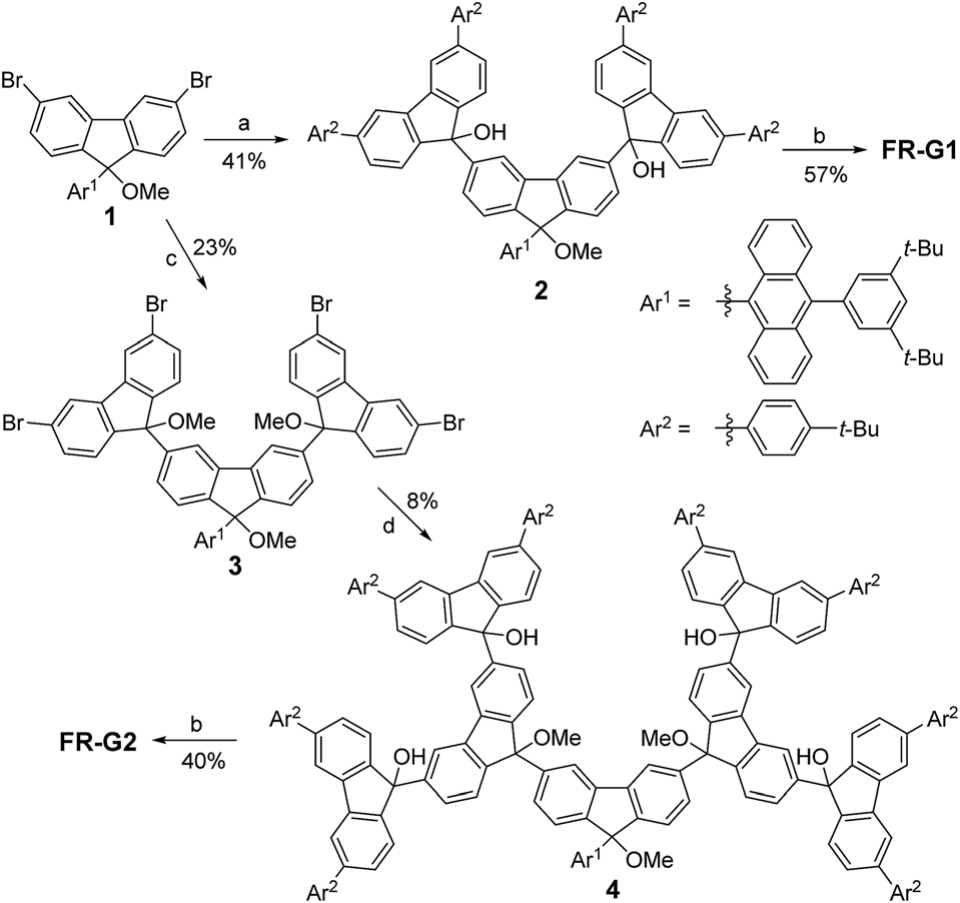
Synthetic routes of **FR-G1** and **FR-G2**. Reagents and conditions: (a) 2.2 equiv. *n*-BuLi, THF, –78 °C, then 2.5 equiv. 3,6-bis(4-*tert*-butylphenyl)-9*H*-fluoren-9-one; (b) SnCl_2_, CH_2_Cl_2_, rt; (c) (i) 2.2 equiv. *n*-BuLi, THF, –78 °C, then 2.2 equiv. 3,6-dibromo-9*H*-fluoren-9-one; (ii) NaH, THF, CH_3_I; (d) 6.0 equiv. *n*-BuLi, THF, –78 °C, then 8.0 equiv. 3,6-bis(4-*tert*-butylphenyl)-9*H*-fluoren-9-one.

The key synthetic strategy toward **FR-G1** and **FR-G2** is to build up the corresponding dendritic precursors with a hydroxy or a methoxy group at the 9-methylene positions, followed by reduction (Scheme 1). A divergent synthetic route was used starting from the 3,6-dibromo-fluorenyl ether **1**.^6^ Lithium–bromine exchange of **1** with 2.2 equivalents *n*-butyllithium followed by quenching with 2.5 equivalents 3,6-bis(4-*tert*-butylphenyl)-9*H*-fluoren-9-one^11^ gave the alcohol precursor **2** in 41% yield. Reduction of **2** by SnCl_2_ in dry dichloromethane (DCM) at room temperature afforded the targeted compound **FR-G1** as a purple solid in 57% yield after purification by normal silica gel chromatography. Compound **FR-G1** is stable, and a half-life time of about 143 h was determined in DCM solution upon exposure to the ambient air and light conditions as monitored by UV-vis-NIR absorption spectroscopy (Fig. S1 in the ESI†). On the other hand, addition of 3,6-dibromo-9*H*-fluoren-9-one into the aryl lithium salt of **1** gave the di-alcohol intermediate, and the hydroxy groups were then protected by methylation with iodomethane to give tri-ether **3**. Similarly, lithium–bromine exchange of **3** with 6.0 equivalents *n*-butyllithium followed by reaction with 8.0 equivalents 3,6-bis(4-*tert*-butylphenyl)-9*H*-fluoren-9-one afforded the precursor **4**, which was carefully purified by preparative gel permeation chromatography. Treatment of compound **4** with SnCl_2_ in dry DCM followed by silica gel column chromatography successfully gave the target compound **FR-G2** as a purple solid in 40% yield. **FR-G2** is also a stable compound but with a slightly shorter half-life time (102 h) in DCM compared to **FR-G1** under the same ambient air and light conditions (Fig. S1 in the ESI†). Due to the existence of unpaired electrons, the aromatic resonances in the ^1^H NMR spectra of **FR-G1** and **FR-G2** are significantly broadened at various temperatures. However, high-resolution mass spectrometry (Fig. S2 and S3 in the ESI†) and high performance liquid chromatography measurements (Fig. S4 and S5 in the ESI†) clearly confirmed the formation of the target compounds with high purity.

### Ground-state electronic structure and polyradical character

**Fig. 2:**
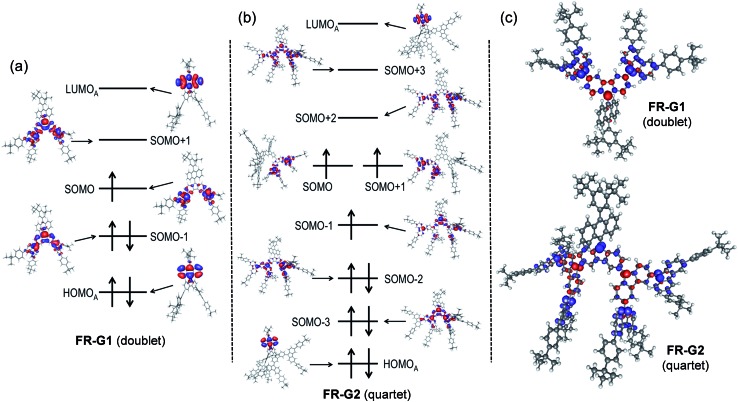
Molecular orbital diagrams of dominant electronic configurations (RAS-SF/6-31G*) (a, b) and spin density distribution (UB3LYP/6-31G*) (c) of **FR-G1** and **FR-G2** in their respective ground states. Subindex A indicates a molecular orbital localized on the anthracene moiety.

The fundamental electronic structure and radical character of **FR-G1** and **FR-G2** were theoretically investigated by the restricted active space spin flip (RAS-SF/6-31G*) method,^12^ a multi-configurational (spin complete) wave function approach that has shown very good results in the description of strongly correlated electron systems,^6^,^7^ and with spin-unrestricted density functional theory (UB3LYP/6-31G*). The calculated electronic energies predict that **FR-G1** has a doublet (D_0_) ground state, with several higher-energy doublet excited states (D_*n*_, *n* = 1–4) and quartet states (Q_*n*_, *n* = 1–4) (Fig. S12 and Table S1 in the ESI†). The energy gap between the ground doublet state and the lowest energy quartet state (Δ*E*_D–Q_) was calculated to be –8.19 kcal mol^–1^ (Table 1). On the other hand, **FR-G2** was predicted to have a quartet ground state with a slightly higher lying doublet excited state (Δ*E*_D–Q_ = +0.78 kcal mol^–1^) (Table 1, Fig. S11 and Tables S2 and S4 in the ESI†). This is reasonable considering that there is minimum of one (for **FR-G1**) or three (for **FR-G2**) unpaired electrons in their AFM coupled resonance forms (Fig. 1). The calculated electronic structures and spin density distribution maps in their respective ground states are shown in Fig. 2. In all cases, the unpaired electron density is delocalized throughout at least two neighbouring fluorenyl units, with the highest density localized at the 9-position carbon centers, indicating moderate AFM exchange coupling between these fluorenyl units. The spin densities are also delocalized throughout the whole branched fluorenyl backbone, indicating two-dimensional π-conjugation. The radical character of their ground-state structures was evaluated by the number of unpaired electrons (*N*_U_)^13^ according to the equation: *N*_U_ = Σ(1 – *abs*(1 – *n*_*i*_)), where {*n*_*i*_} are the natural occupation numbers from the one-particle density matrix. The *N*_U_ value was calculated to be 1.82 for **FR-G1** and 5.23 for **FR-G2** (Table 1). Significant electronic occupancies were calculated for the lowest unoccupied natural orbitals (SONO + *i*, *i* = 1,2,3,…) of **FR-G1** and **FR-G2** (Table 1 and Tables S1, S3 in the ESI†), which should directly correspond to Yamaguchi's polyradical character indices.^14^ Accordingly, **FR-G1** has a moderate triradical character (*y*_0_ = 0.37), while **FR-G2** has large triradical character (*y*_0_ = 1.0), moderate pentaradical character (*y*_1_ = 0.58), and moderate heptaradical character (*y*_2_ = 0.50). All these calculations suggest a moderate AFM exchange interaction between the fluorenyl units in both dendrons.

**Table 1:** Calculated (RAS-SF/6-31G*) energy gap between the lowest doublet state and the lowest quartet state (Δ*E*_D–Q_) for **FR-G1** and **FR-G2**, and the unpaired electron numbers (*N*_U_) and the occupation numbers of SONO + *i* (*i* = 1, 2, 3) in their respective ground states (doublet for **FR-G1** and quartet for **FR-G2**)

	Δ*E*_D–Q_ (kcal mol^–1^)	*N*_U_	*n*(SONO + 1)	*n*(SONO + 2)	*n*(SONO + 3)
**FR-G1**	–8.19	1.82	0.37	—	—
**FR-G2**	+0.78	5.23	1.00	0.58	0.50

### Magnetic properties

**Fig. 3:**
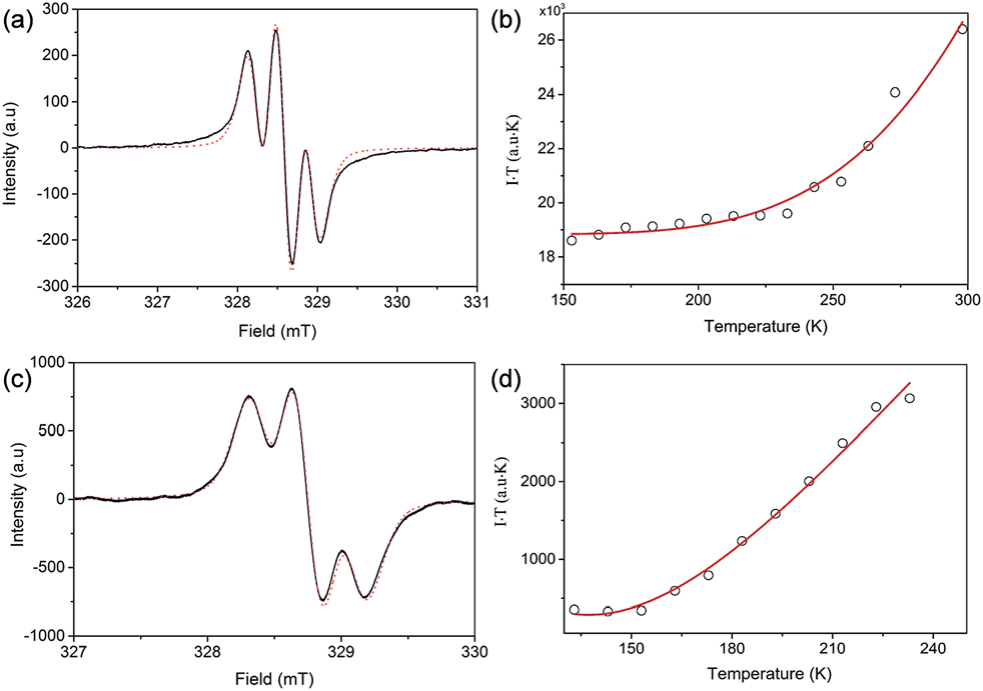
Measured (solid line) and fitted (dash line) ESR spectra of **FR-G1** (a) and **FR-G2** (b) in DCM at 298 K. Measured (circle) and fitted (solid line) *IT*–*T* curves based on the VT ESR measurements of **FR-G1** (c) and **FR-G2** (d) in the solid state.

Both **FR-G1** and **FR-G2** showed strong electron spin resonance (ESR) signals in DCM solution with the same *g*_e_ value of 2.0026, and the ESR spectra can be well fitted by considering the spin-nucleus hyperfine coupling (Fig. 3a and c and S6 in the ESI†). Compared with fluorenyl monoradicals,^6^,^8^ the ESR spectra of both compounds are broadened, indicating a moderate spin–spin exchange interaction between the fluorenyl units. **FR-G2** exhibited a broader ESR spectrum compared to **FR-G1** presumably due to more extended spin delocalization. Variable-temperature ESR measurements were conducted for the powder form, and in both cases, the product of ESR intensity (*I*) and temperature (*T* in K) increases with temperature (Fig. 3b and d and S7 in the ESI†), correlating to a thermal population from the ground state to higher energy excited states. Fitting of the ESR data by using a trimer model for **FR-G1** and a simplified pentanuclear model for **FR-G2** gave a Δ*E*_D–Q_ value of –3.9 kcal mol^–1^ and +0.2 kcal mol^–1^, respectively (see details in the ESI†). Therefore, the ground state of **FR-G1** is a doublet while **FR-G2** has a quartet ground state, in agreement with the theoretical predictions.

### Optical and electrochemical properties

**Fig. 4:**
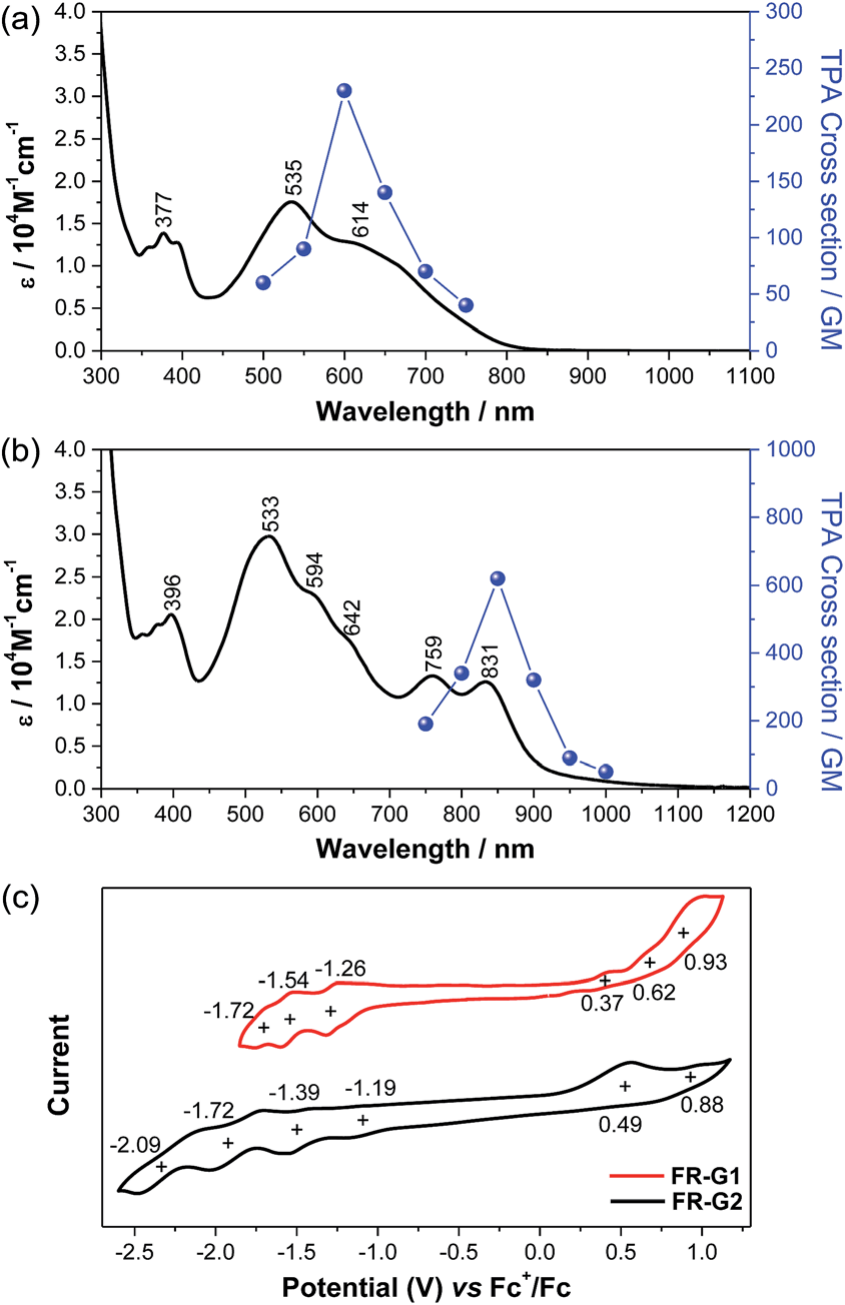
One-photon and two-photon absorption spectra of **FR-G1** (a) and **FR-G2** (b) in DCM. TPA spectra are plotted at *λ*_ex_/2. (c) Cyclic voltammograms of **FR-G1** and **FR-G2** measured in DCM.

**FR-G1** in DCM shows an intense absorption band in the vis-NIR region extending up to 850 nm, with maximum (*λ*_max_) at 535 nm (Fig. 4a), indicating significant AFM exchange interaction (or π-conjugation) among the three radicals. On the other hand, the fluorenyl radical monomer **FR-G0** without intramolecular AFM coupling exhibits a long and weak absorption band up to 1050 nm.^6^**FR-G2** in DCM displays a new moderate-intense band with peaks at 759 and 831 nm in addition to the intense band centered at 533 nm, and the absorption is extended up to 1150 nm (Fig. 4b), which can be explained by multiple AFM coupling between the fluorenyl radicals. The optical energy gap (*E*optg) of **FR-G1** and **FR-G2** was estimated to be 1.56 eV and 1.12 eV, respectively, from the lowest energy absorption onset. Assignments of the absorption bands of **FR-G1** and **FR-G2** in terms of orbital transitions can be found in the ESI (Tables S5, S6, Fig. S13 and S14†).

Open-shell singlet diradicaloids and polyradicaloids having moderate bonding of the frontier π-electrons usually show enhanced two-photon absorption (TPA).^15^ Hence, TPA properties of **FR-G1** and **FR-G2** were probed by using the open-aperture Z-scan method in the wavelength range where one-photon absorption contribution is negligible (Fig. 4a and b and S8 in the ESI†). **FR-G1** exhibited a maximum TPA cross-section value (*σ*_max_^(2)^) of 230 GM at 1200 nm, while **FR-G2** showed a largely increased *σ*_max_^(2)^ value of 620 GM at 1700 nm due to more extended 2D π-conjugation *via* multiple intramolecular AFM exchange interactions. Both *σ*_max_^(2)^ values are larger than typical closed-shell π-conjugated systems in a similar size. Femtosecond transient absorption (TA) measurements were conducted to investigate their excited-state dynamics (Fig. S9 in the ESI†). According to the kinetic plots of ground-state bleaching domains, it was observed that **FR-G1** and **FR-G2** were fitted with double exponential functions in TA spectra (Fig. S9 in the ESI†). The fast decay time of a few picoseconds can be attributed as an excited-state lifetime, whereas the longer decay time corresponds to the structural relaxation. Due to the increased polyradical character originating from AFM exchange interaction, the lifetime of **FR-G2** decreased from 3 ps to 1 ps compared to **FR-G1**.

**FR-G1** showed three irreversible oxidation waves with half-wave potential *E*ox1/2 = 0.37, 0.62 and 0.93 V (*vs.* Fc^+^/Fc) and three quasi-reversible reduction waves with *E*red1/2 = –1.26, –1.54 and –1.72 V during the cyclic voltammetry and pulse voltammetry measurement (Fig. 4c and S10 in the ESI†). The HOMO and LUMO energy levels are estimated to be –5.08 and –3.77 eV from the onset potential of the first oxidation and reduction wave, respectively, and the corresponding electrochemical energy gap (*E*ECg) is 1.31 eV. **FR-G2** exhibited two oxidation waves at *E*ox1/2 = 0.49 and 0.88 V and four reduction waves at *E*red1/2 = –1.19, –1.39, –1.72 and –2.09 V (Fig. 4c). The HOMO and LUMO energy levels of **FR-G2** were estimated to be –4.96 and –3.93 eV, with a smaller *E*ECg value of 1.03 eV. Therefore, with increasing molecular size, the HOMO increases and the LUMO decreases. The trend of the electrochemical energy gap is in consistent with the observed optical energy gap, and the decrease of band gap from **FR-G1** to **FR-G2** can be simply explained by more extended 2D π-conjugation in **FR-G2**. Spectro-electrochemical studies reveal that **FR-G1** can be fully oxidized to its trications with *λ*_max_ at 1088 nm and fully reduced to its trianions with *λ*_max_ at 378 nm (Fig. S11 in the ESI†). **FR-G2** can be oxidized to trications (*λ*_max_ = 1090 nm) and fully reduced to hepta-anions (*λ*_max_ ≈ 376 nm). The multiple redox behaviour is due to their polyradical character and 2D π-conjugation, which can stabilize multiple charges.

## Conclusions

In summary, stable fluorenyl radical dendrons up to the second generation were successfully synthesized. The moderate intramolecular AFM coupling between the fluorenyl radicals results in two-dimensionally π-conjugated structures with polyradical character. Due to the more extended π-conjugation and polyradical character, the second generation dendron **FR-G2** exhibited smaller energy gap, larger TPA cross-section, and shorter excited state lifetime compared to the first generation dendron **FR-G1**. Both compounds showed small electrochemical energy gaps and multiple accessible redox waves. Our molecules represent the first class of two-dimensionally AFM coupled dendritic polyradicaloids.

## Conflicts of interest

There are no conflicts to declare.

## Supplementary Material

Supplementary information
