# Supplementary material for: Stable 2D anti-ferromagnetically coupled fluorenyl radical dendrons
Source: Chem Sci. 2018 Feb 28;9(13):3395–400. doi: 10.1039/c7sc05493a (PMC5933225; doi:10.1039/c7sc05493a)
Supplement: Supplementary file 1 [file SC-009-C7SC05493A-s001.pdf]

Electronic Supplementary Material (ESI) for Chemical Science.
This journal is © The Royal Society of Chemistry 2018

Electronic Supplementary Information (ESI) for

Stable 2D anti-ferromagnetically coupled fluorenyl radical dendrons

Jian Wang,[a] Gakhyun Kim,[b] María Eugenia Sandoval-Salinas,[cd] Hoa Phan,[a] Tullimilli Y. Gopalakrishna,[a] Xuefeng Lu,[a] David Casanova,[,c] Dongho Kim,[*,b] and Jishan Wu[*,a]*

[a]Department of Chemistry, National University of Singapore, 3 Science Drive 3, 117543, Singapore

[b]Department of Chemistry, Yonsei University, Seoul 120-749, Korea

[c]Kimika Fakultatea, Euskal Herriko Unibertsitatea & Donostia International Physics Center, Paseo Manuel de Lardizabal, 4, 20018, Donostia-San Sebastián, Euskadi, Spain

[d]Departament de Ciència de Materials i Química Física, Institut de Química Teòrica i Computacional (IQTCUB), Universitat de, Barcelona, Martí i Franquès 1-11, Barcelona 08028, Spain

Table of Content

1. Experimental section……………………………………………………………………S2

1.1 General…………………………………………………………………………...S2

1.2 Synthetic procedures and characterization data…………………………………….S5

2. Additional spectra……..……………..……………………………….……….......................S9

3. Electronic structure calculations….....……………………………………………………S15

4. Additional NMR and mass spectra……………………………….…………….....……S22

5. References………………………………………………………………………..S26

1. Experimental Section

1.1 General

All reagents and starting materials were obtained from commercial suppliers and used without further purification unless otherwise noted. Anhydrous toluene and THF were distilled from sodium-benzophenone immediately prior to use. Anhydrous dichloromethane (DCM) was distilled from CaH_2. All reaction conditions dealing with air- and moisture sensitive compounds were carried out in a dry reaction vessel under an Argon atmosphere. 9-(3,5-di-*tert*-butylphenyl)-10-(3,6-dibromo-9-methoxy-9H-fluoren-9-yl)anthracene (**1**)[1] and 3,6-bis(4-*tert*-butylphenyl)-9H-fluoren-9-one[2] were prepared according to the literatures. The ^{1}H NMR and ^{13}C NMR spectra were recorded in solution of $CDCl_3$ or CD_2Cl_2 on Bruker DPX 300, DPX 400 or DRX 500 NMR spectrometers. All chemical shifts are quoted in ppm, relative to tetramethylsilane, using the residual solvent peak as a reference standard. The following abbreviations were used to explain the multiplicities: s = singlet, d = doublet, m = multiplet. Atmospheric Pressure Chemical Ionization Mass Spectrometry (APCI MS) measurements were performed on a Finnigan TSQ 7000 triple stage quadrupole mass spectrometer. EI mass spectra were recorded on Agilent5975C DIP/MS mass spectrometer. UV-vis-NIR absorption spectra was recorded on a Shimadzu UV-1700/UV-3600 spectrophotometer. Cyclic voltammetry measurements were performed in dry DCM on a CHI 620C electrochemical analyzer with a three-electrode cell, using 0.1 M Bu_4NPF_6 as supporting electrolyte, AgCl/Ag as reference electrode, gold disk as working electrode, Pt wire as counter electrode, and scan rate at 50 mV/s. The potential was externally calibrated against the ferrocene/ferrocenium couple. Spectroelectrochemical measurements were conducted by using a commercially available spectrochemical cell with platinum as working electrode and the potential was controlled by an Autolab Potentiostat.

Continuous wave X-band ESR spectra were obtained with a JEOL (FA200) spectrometer using a variable temperature liquid nitrogen cryostat. The variable temperature ESR data of **FR-G1** was fitted by equation (1) based on a linear trimer model:

The spin Hamiltonian can be written as:

$$H = -2J(S_1S_2 + S_2S_3)$$

Then the energies

$E(S_T,S_{13},S_{24}) = -J[S_T(S_T+1) - S_{13}(S_{13} + 1)]$

where $S_T = S_1 + S_2 + S_3$; $S_{13} = S_1 + S_3$.

Using the energies deduced from the above to the Van Vleck's equation,[3] the product of the ESR signal intensity and temperature can be expressed as:

$$I\cdot T = \frac{aN\beta^2 g^2}{3k_B}\,\frac{3/2exp(2J/k_BT)+ 15exp(3J/k_BT)+ 3/2}{2exp(2J/k_BT)+ 4exp(3J/k_BT)+ 2} \qquad (1)$$

where, $-2J$ is correlated to the excitation energy from the doublet ground state to the lowest quartet excited state ($\Delta E_{\text{D-Q}}$)

The variable temperature ESR data of **FR-G2** was fitted by equation (2) based on a pentanuclear model:

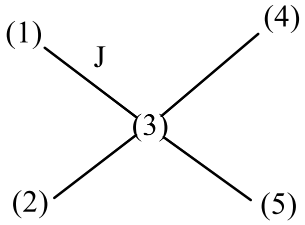

The spin Hamiltonian can be written as:

$H = -2J(S_1S_3 + S_2S_3 + S_3S_4 + S_3S_5)$

Then the energies

$E_i = E(S_{T},S') = -J[S_T(S_T+1) - S'(S' + 1)]$

where $S_T = S_1 + S_2 + S_3 + S_4 + S_5$; $S' = S_1 + S_2 + S_4 + S_5$;

and J is the energy gap between the quartet ground state to the lowest doublet excited state.

Using the energies deduced from the above to the Van Vleck's equation,[3] the product of the ESR signal intensity and temperature can be expressed as:

$$I{\cdot}T = \frac{aN\beta^2 g^2}{k_B} \frac{\sum_{i=1}^{5}\sum_{M_S=-S_T}^{M_S=S_T} M_S^2 exp(-E_i/k_B T)}{\sum_{i=1}^{5}(2S_T+1) exp(-E_i/k_B T)} \qquad (2)$$

The femtosecond time-resolved transient absorption (*fs*-TA) spectrometer consists of an optical parametric amplifier (OPA; Palitra, Quantronix) pumped by a Ti: sapphire regenerative amplifier system (Integra-C, Quantronix) operating at 1 kHz repetition rate and an optical detection system. The generated OPA pulses have a pulse width of ~ 100 fs and an average power of 1 mW in the range of 280-2700 nm, which are used as pump pulses. White light continuum (WLC) probe pulses were generated using a sapphire window (3 mm thick) by focusing a small portion of the fundamental 800 nm pulses, which was picked off by a quartz plate before entering the OPA. The time delay between pump and probe beams was carefully controlled by making the pump beam travel along a variable optical delay (ILS250, Newport). Intensities of the spectrally dispersed WLC probe pulses are monitored by a High Speed Spectrometer (Ultrafast Systems) for both visible and near-infrared measurements. To obtain the time-resolved transient absorption difference signal (ΔA) at a specific time, the pump pulses were chopped at 500 Hz and absorption spectra intensities were saved alternately with or without pump pulse. Typically, 4000 pulses excite the samples to obtain the *fs*-TA spectra at each delay time. The polarization angle between pump and probe beam was set at the magic angle (54.7°) using a Glan-laser polarizer with a half-wave retarder in order to prevent polarization-dependent signals. Cross-correlation *fwhm* in pump-probe experiments was less than 200 fs and chirp of WLC probe pulses was measured to be 800 fs in the 400-800 nm region. To minimize chirp, all reflection optics in the probe beam path and a quartz cell of 2 mm path length were used. After *fs*-TA experiments, the absorption spectra of all compounds were carefully examined to detect if there were artifacts due to degradation and photo-oxidation of samples. The three-dimensional data sets of ΔA versus time and wavelength were subjected to singular value decomposition and global fitting to obtain the kinetic time constants and their associated spectra using Surface Xplorer software (Ultrafast Systems).

The two-photon absorption spectrum was measured in the NIR region using the open-aperture Z-scan method with 130 fs pulses from an optical parametric amplifier (Light Conversion, TOPAS) operating at a repetition rate of 1 kHz generated from a Ti: sapphire regenerative amplifier system (Spectra-Physics, Hurricane). After passing through a 10 cm focal length lens, the laser beam was focused and passed through a 1 mm quartz cell. Since the position of the sample

cell could be controlled along the laser beam direction (z axis) using the motor controlled delay stage, the local power density within the sample cell could be simply controlled under constant laser intensity. The transmitted laser beam from the sample cell was then detected by the same photodiode as used for reference monitoring. The on-axis peak intensity of the incident pulses at the focal point, I_0, ranged from 40 to 60 GW cm^{-2}. For a Gaussian beam profile, the nonlinear absorption coefficient can be obtained by curve fitting of the observed open-aperture traces $T(z)$ with the following equation:

$$T(z)=1-\frac{\beta I_0(1-e^{-\alpha_0 l})}{2\alpha_0[1+(z/z_0)^2]}$$

where α_0 is the linear absorption coefficient, l is the sample length, and $z0$is the diffraction length of the incident beam. After the nonlinear absorption coefficient has been obtained, the TPA cross section $\sigma^{(2)}$of one solute molecule (in units of GM, where 1 GM = 10^{-50} cm^4 s $photon^{-1}$ $molecule^{-1}$) can be determined by using the following relationship:

$$\beta=\frac{10^{-3}\sigma^{(2)}N_A d}{h\nu}$$

Where N_A is the Avogadro constant, d is the concentration of the compound in solution, h is the Planck constant, and v is the frequency of the incident laser beam.

1.2 Synthetic procedures and characterization data

3,3'',6,6''-Tetrakis(4-*tert*-butylphenyl)-9'-(10-(3,5-di-*tert*-butylphenyl)anthracen-9-yl)-9'-methoxy-9H,9'H,9''H-[9,3':6',9''-terfluorene]-9,9''-diol (**2**)

Under argon atmosphere, a solution of compound **1** (1.0 g, 1.40 mmol) in dry THF (100 mL) was cooled to -78 °C and then *n*-BuLi (1.6 M in n-hexane, 1.9 mL, 3.08 mmol) was added slowly. The solution was stirred at -78 °C for 1 h and then 3,6-bis(4-*tert*-butylphenyl)-9H-fluoren-9-one (1.6 g, 3.5 mmol) was added, the mixture was slowly warmed to room temperature and stirred overnight. The reaction was quenched by water, the mostly THF was removed and extracted by DCM for three times. The combined organic solution was washed with sodium chloride solution and dried over anhydrous sodium sulfate. After removal of the solvent, the residue was purified by flash column chromatography (silica gel, Hexane/DCM/EA = 40:10:1), white solid alcohol

intermediate compound **2** was obtained in 41% yield (830 mg). ^{1}H NMR (300 MHz, CD_2Cl_2, δ ppm): 9.55 (d, 1H, *J* = 9.2 Hz), 8.14 (s, 2H), 8.01 (s, 4H), 7.69-7.59 (m, 10H), 7.58-7.48 (m, 14H), 7.46-7.43 (m, 5H), 7.42-7.28 (m, 2H), 7.24-7.21 (m, 4H), 7.15-7.12 (m, 2H), 7.06-6.99 (m, 2H), 6.75-6.70(m, 1H), 1.38-1.36(s, 54H). ^{13}C NMR (75 MHz, CD_2Cl_2, δ ppm): 151.28, 151.15, 150.11, 150.04, 148.35, 145.31, 142.86, 141.19, 140.79, 140.68, 138.43, 132.65, 132.09, 131.65, 130.67, 129.00, 128.02, 127.24, 126.54, 126.29, 126.11, 125.95, 125.38, 124.55, 124.36, 123.56, 121.50, 119.31, 118.34, 91.57, 83.90, 51.08, 35.33, 34.94, 31.75, 31.56. HRMS (ESI, *m*/*z*): $[M+Na]^+$ calcd. for $C_{108}H_{104}NaO_3$: *m*/*z* = 1471.7878; found, 1471.7912.

Synthesis of FR-G1

Under argon atmosphere, compound **2** (100 mg, 0.072 mmol) was dissolved in a solution of dry DCM (20 mL), then $SnCl_2$ (137 mg, 0.72 mmol) was added. The mixture was stirred at room the temperature overnight. The solvent was removed under reduced pressure at room temperature. Then the residue was purified by flash column chromatography (silica gel, DCM/Hexane = 1:20). Purple solid **FR-G1** was obtained in 57% yield (57 mg). The ^{1}H NMR spectra at variable temperature was significantly broadened due to its paramagnetic nature. MALDI-TOF Mass: *m*/*z* 1384.1, calc. 1384.8. HRMS (APCI, *m*/*z*): $[(M+H)^+]$ calcd for $C_{107}H_{100}$, 1384.7820; found, 1384.7821 (Fig. S2). The purity was further confirmed by HPLC (Fig. S4).

3,3'',6,6''-Tetrabromo-9'-(10-(3,5-di-*tert*-butylphenyl)anthracen-9-yl)-9,9',9''-trimethoxy-9H,9'H,9''H-9,3':6',9''-terfluorene (**3**)

Under argon atmosphere, a solution of compound **1** (5.0 g, 6.9 mmol) in dry THF (350 mL) was cooled to -78 °C and then *n*-BuLi (1.6 M in n-hexane, 9.5 mL, 15.2 mmol) was added slowly. The solution was stirred at -78 °C for 1 h and then 3,6-dibromo-9H-fluoren-9-one (5.1 g, 15.2 mmol) was added, the mixture was slowly warmed to room temperature and stirred overnight. The reaction was quenched by water, the mostly THF was removed and extracted by DCM for three times. The combined organic solution was washed with sodium chloride solution and dried over anhydrous sodium sulfate. After removal of the solvent, the residue was purified by flash column chromatography (silica gel, Hexane/DCM/EA = 40:10:1), and pale yellow solid alcohol intermediate compound of **3** was obtained. It was used directly for next step. Then intermediate alcohol (2 g, 1.6mmol) was dissolved in dry THF (50 mL) under argon atmosphere, sodium

hydride (60% oil suspension, 140 mg, 3.5 mmol) was added into the system at 0 °C, and the mixture was stirred for overnight at room temperature under argon. The reaction was quenched by water, the mostly THF was removed and extracted by DCM for three times. The combined organic solution was washed with sodium chloride solution and dried over anhydrous sodium sulfate. After removal of the solvent, the residue was purified by flash column chromatography (silica gel, Hexane/DCM/EA = 40:10:1), pale yellow solid **3** was obtained in 23% yield over two steps (1.98 g). ^{1}H NMR (300 MHz, CD_2Cl_2, δ ppm): 9.51 (d, 1H, *J* = 9.2 Hz), 8.00 (d, 2H, *J* = 1.4 Hz), 7.87 (d, 4H, *J* = 1.6 Hz), 7.68 (d, 1H, *J* = 8.0 Hz), 7.59 (t, 1H, *J* = 1.8 Hz), 7.52-7.42 (m, 6H), 7.35-7.27 (m, 1H), 7.27-7.19 (m, 6H), 7.15 (d, 2H, *J* = 8.0 Hz), 7.07-6.99 (m, 1H), 6.96-6.85 (m, 3H), 6.66-6.61 (m, 1H), 2.99 (s, 6H), 2.87 (s, 3H), 1.37 (s, 18H). ^{13}C NMR (75 MHz, CD_2Cl_2, δ ppm): 151.17, 148.52, 146.61, 146.49, 144.16, 142.39, 142.22, 141.04, 140.75, 138.68, 132.29, 131.64, 130.64, 128.85, 128.14, 127.86, 127.41, 126.41, 126.09, 126.04, 125.91, 124.45, 124.24, 123.92, 123.86, 123.46, 121.54, 118.31, 91.46, 89.04, 51.78, 51.10, 35.34, 31.76. HRMS (ESI, *m/z*): $[M+Na]^+$ calcd. for $C_{70}H_{56}Br_4NaO_3$: *m/z* = 1283.0885; found, 1283.0887.

Precursor of FR-G2 (compound 4)

Under argon atmosphere, a solution of compound **3** (5.0 g, 4.0 mmol) in dry THF (500 mL) was cooled to -78 °C and then *n*-BuLi (1.6 M in n-hexane, 15 mL, 24.0 mmol) was added slowly. The solution was stirred at -78 °C for 2 h and then 3,6-bis(4-*tert*-butylphenyl)-9H-fluoren-9-one (14 g, 32.0 mmol) was added, the mixture was keep at -78 °C for 8 hours then slowly warmed to room temperature and stirred overnight. The reaction was quenched by water, the mostly THF was removed and extracted by DCM for three times. The combined organic solution was washed with sodium chloride solution and dried over anhydrous sodium sulfate. After removal of the solvent, the residue was purified by flash column chromatography (silica gel, Hexane/DCM/EA = 20:10:1), the crude product of **4** as a pale yellow solid alcohol was obtained. The crude product was further purified by recycling preparative GPC to give the pure compound **4** in 8% yield (872 mg). The low yield is due to the loss of sample during the isolation. ^{1}H NMR (400 MHz, CD_2Cl_2, δ ppm): 9.52 (d, 2H, *J* = 8.5 Hz), 8.55 (d, 0.5H, *J* = 7.6 Hz), 8.27-7.94 (m, 43.5H), 7.82 (d, 2H, *J* = 8.1 Hz), 7.70-7.58 (m, 58H), 7.55-7.49 (m, 76H), 7.47-7.38 (m, 24H), 7.34-7.30 (m, 5H), 7.25-7.23 (m, 22H), 7.16 (d, 2H, *J* = 9.1 Hz), 7.11 (d, 4H, *J* = 8.0 Hz), 6.99-6.84 (m, 12H), 6.68-6.55 (m, 2.5H), 6.24 (d, 0.5H, *J* = 7.6 Hz), 2.99 (s, 18H), 2.85 (s, 6H), 2.78 (s, 3H), 2.65 (br s, 12H). ^{13}C NMR (75

MHz, CD_2Cl_2, δ ppm): 151.21, 151.08, 150.90, 149.93, 148.03, 146.78, 146.72, 145.30, 142.74, 141.81, 141.72, 141.05, 140.65, 138.80, 138.36, 132.85, 131.98, 131.56, 130.59, 129.08, 128.89, 127.93, 127.22, 126.27, 125.96, 125.78, 125.46, 124.48, 124.22, 123.52, 122.65, 121.42, 119.23, 118.61, 117.88, 91.42, 89.13, 83.81, 51.70, 50.96, 50.32, 35.45, 35.33, 34.91, 31.79, 31.71, 31.57. HRMS (APCI, *m*/*z*): $[M]^+$ calcd. for $C_{202}H_{188}O_7$: *m*/*z* = 2725.4350; found, 2725.4403.

Synthesis of FR-G2

Under argon atmosphere, compound **4** (100 mg, 0.037 mmol) was dissolved in a solution of dry DCM (20 mL), then $SnCl_2$ (139 mg, 0.73 mmol) was added. The mixture was stirred at room the temperature overnight. The solvent was removed under reduced pressure at room temperature. Then the residue was purified by flash column chromatography (silica gel, DCM/Hexane = 1:20). Purple solid **FR-G2** was obtained in 40% yield (38 mg). The ^{1}H NMR spectra at variable temperature was significantly broadened due to its paramagnetic nature. MALDI-TOF Mass: *m*/*z* 2564.7, calc. 2564.4. HRMS (MALDI, *m*/*z*): $[(M+H)^+]$ calcd for $C_{199}H_{176}$, 2565.3767; found, 2565.3889 (Fig. S3). The purity was further confirmed by HPLC (Fig. S5).

2. Additional spectra data

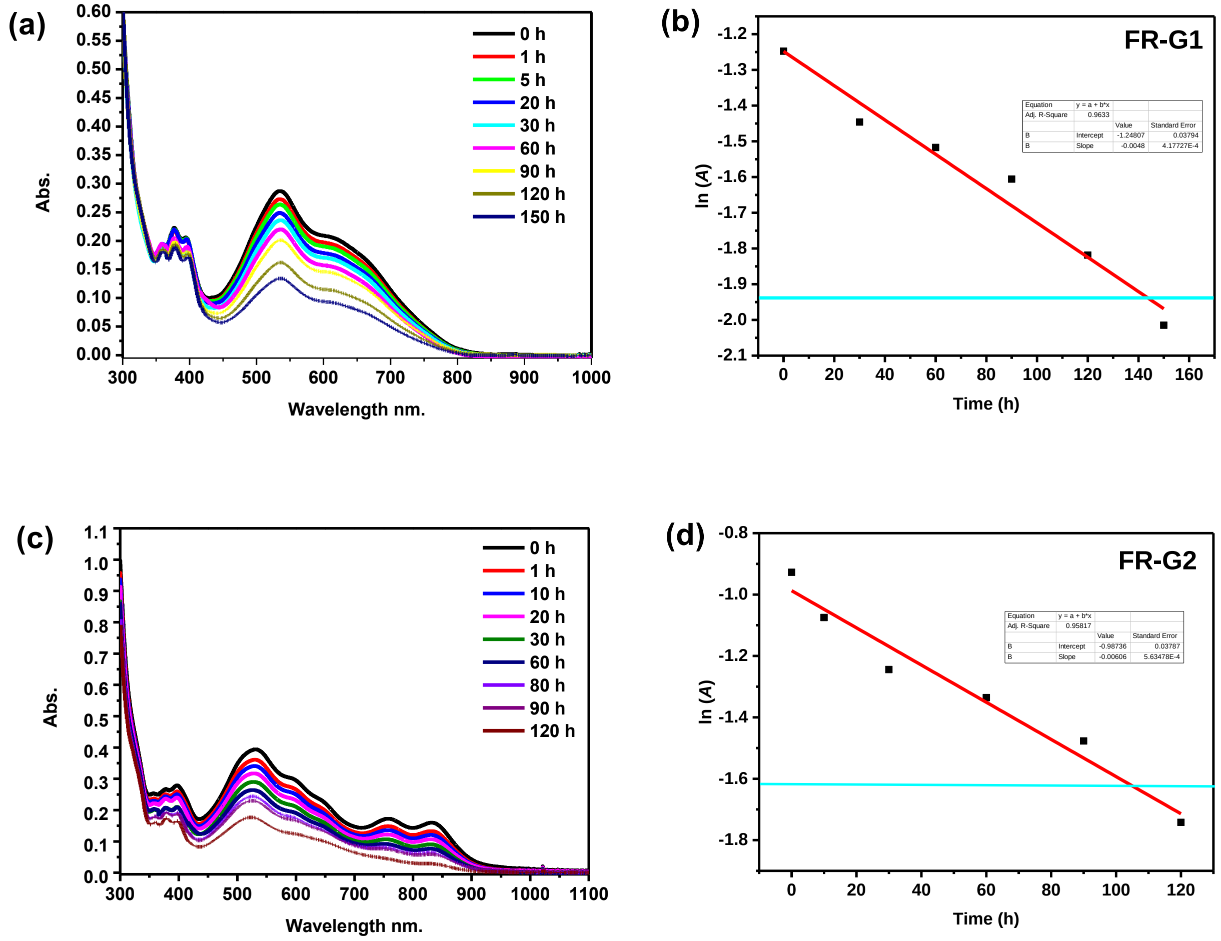

Fig. S1 Absorption spectra of **FR-G1** (a) and **FR-G2** (c) in DCM recorded at different time when exposure to the ambient air and light conditions, and plots of the absorbance (*A*) of **FR-G1** (b) at 534 nm and **FR-G2** (d) at 532 nm with time.

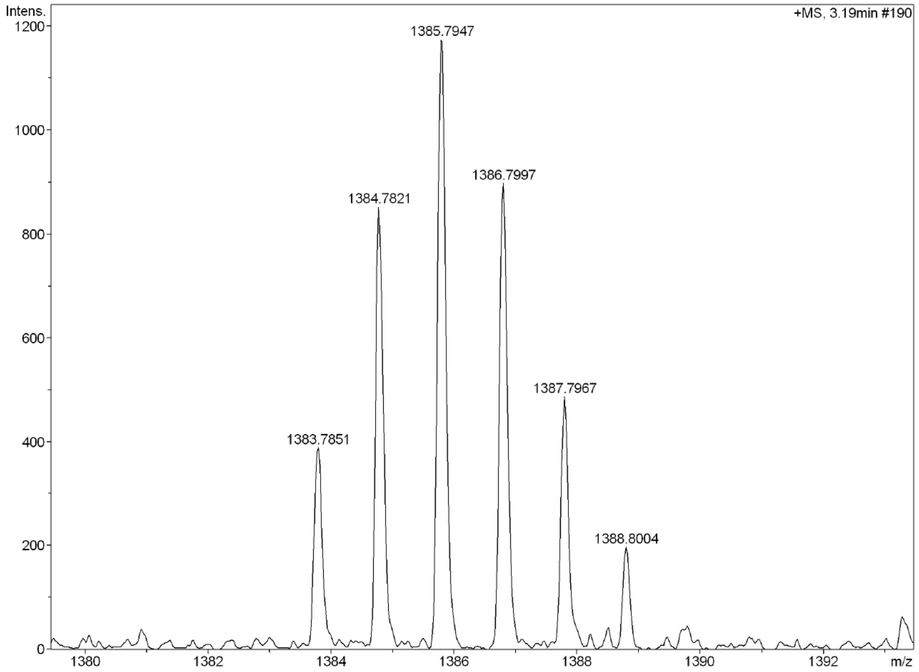

Fig. S2 HR mass spectrum (APCI) of **FR-G1**.

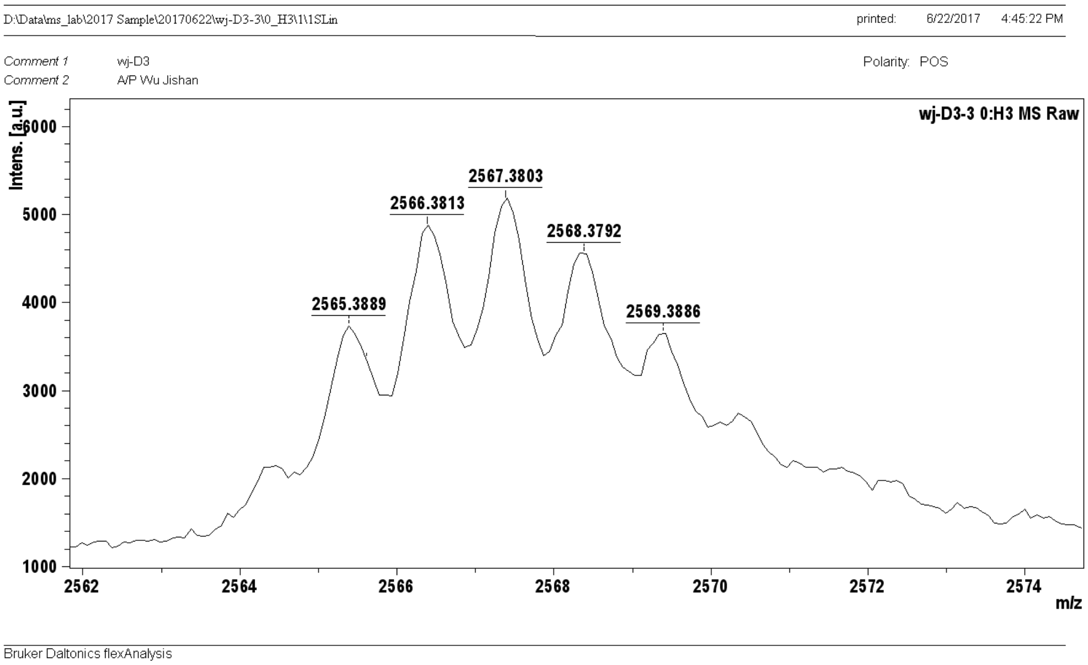

Fig. S3 HR mass spectrum (MALDI) of the **FR-G2**.

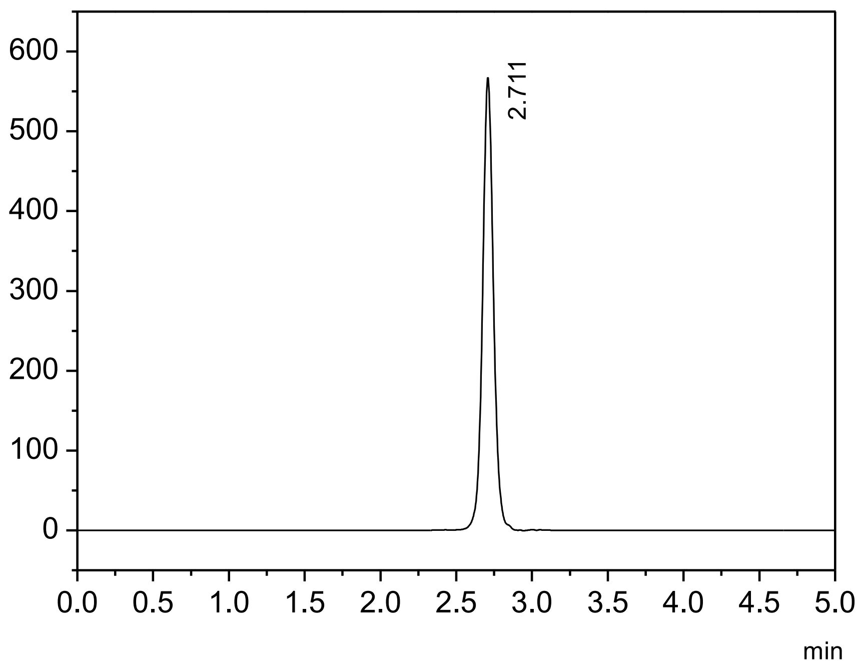

Fig. S4 Representative HPLC curve of compound **FR-G1**. Inertsil C8-3 column (5μm, 4.6 X 250 mm), CH_3CN/THF = 30/70 as eluent, flow rate 1 mL min^{-1}, and detection wavelength is 533 nm.

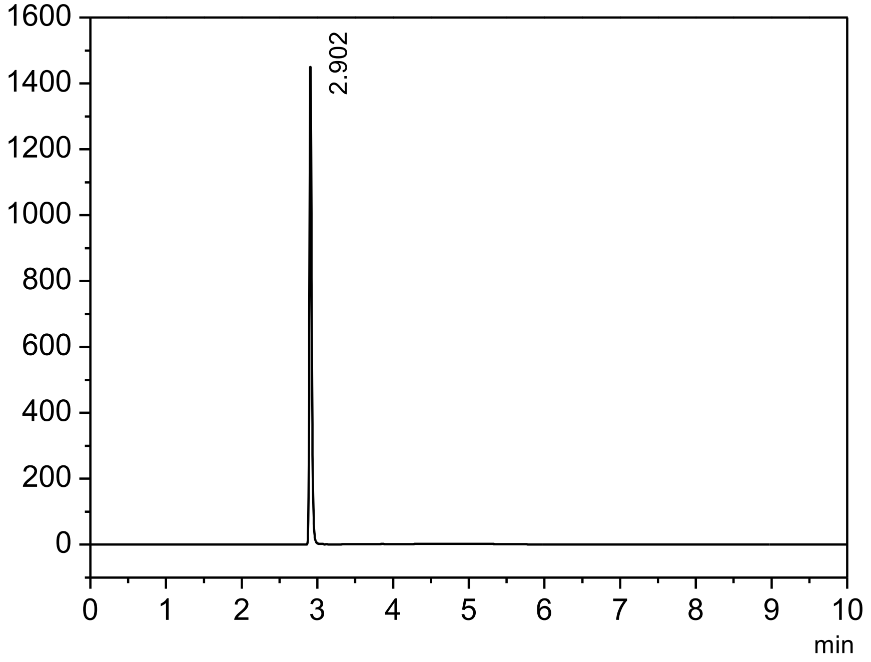

Fig. S5 Representative HPLC curve of compound **FR-G2**. Inertsil C8-3 column (5 μm, 4.6 X 250 mm), CH_3CN/THF = 50/50 as eluent, flow rate 1 mL min^{-1}, and detection wavelength is 533 nm.

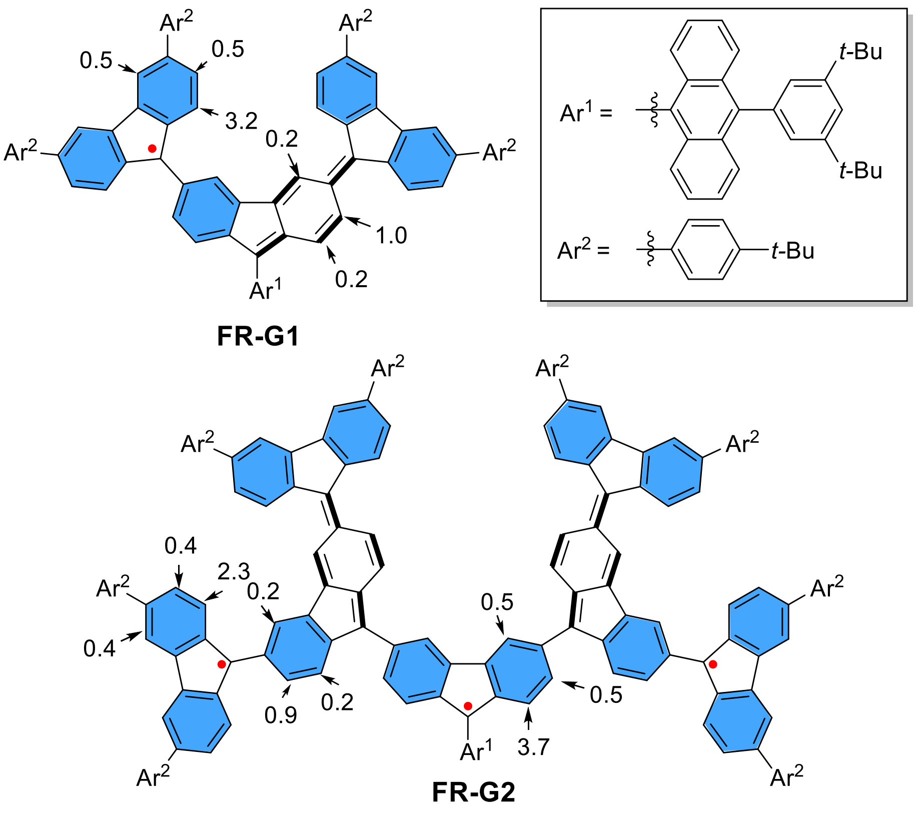

Fig. S6 Hyperfine coupling constants (in Gauss) used for the ESR simulation of **FR-G1** and **FR-G2** in Fig.s 4a/c.

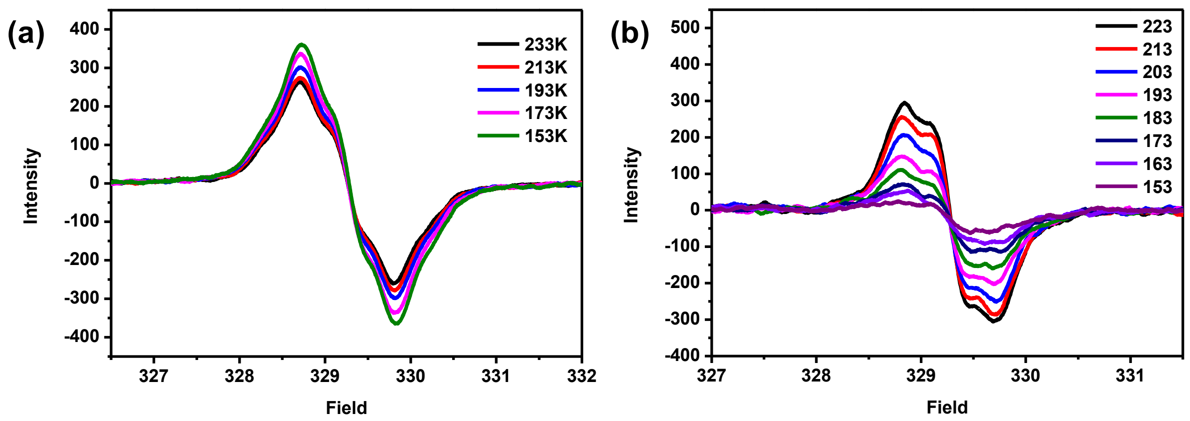

Fig. S7 VT ESR spectra of the compounds **FR-G1** (a) and **FR-G2** (b) in solid.

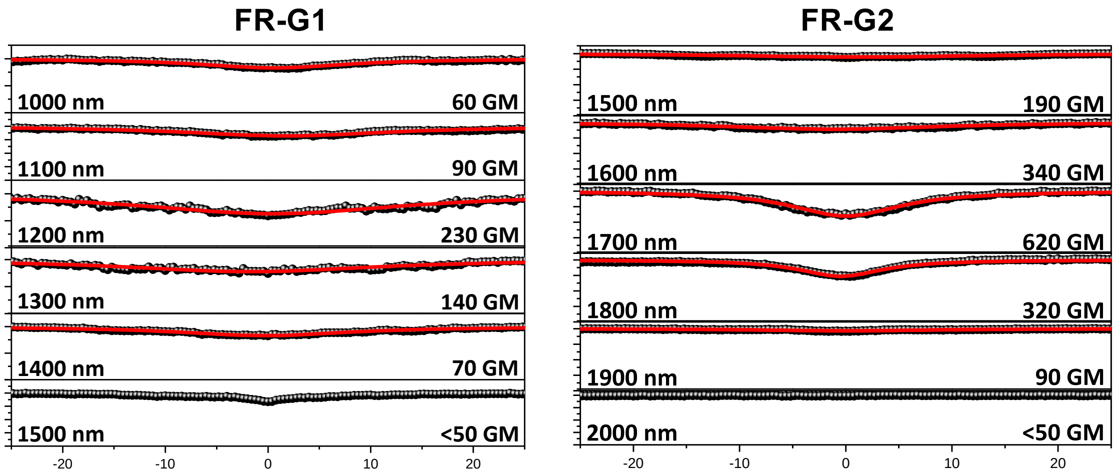

Fig. S8 Z-scan curves of **FR-G1** and **FR-G2** recorded in DCM. Z-scan curves measured by photoexcitation in the range from 1000 to 2000 nm.

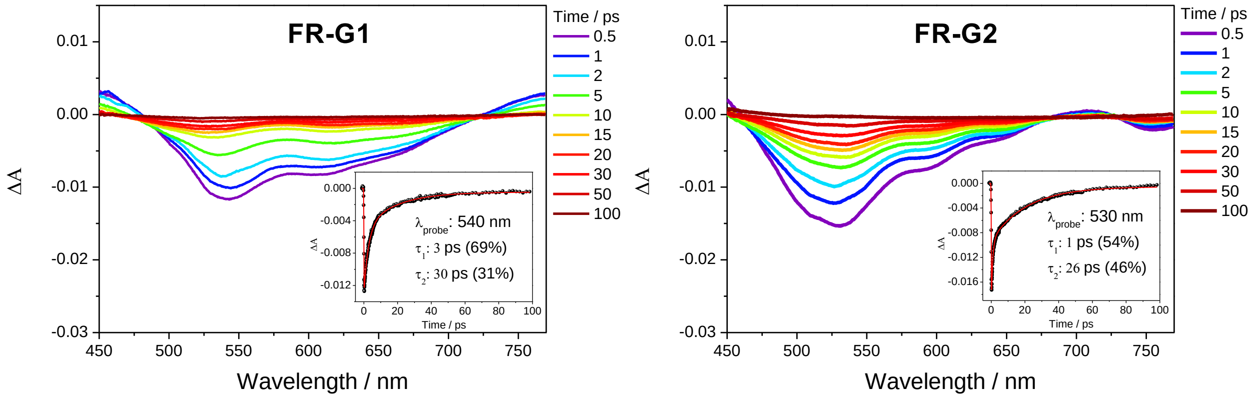

Fig. S9 Transient absorption spectra of **FR-G1** and **FR-G2** recorded in DCM. Inset are the decay curves with time and fitted lifetimes.

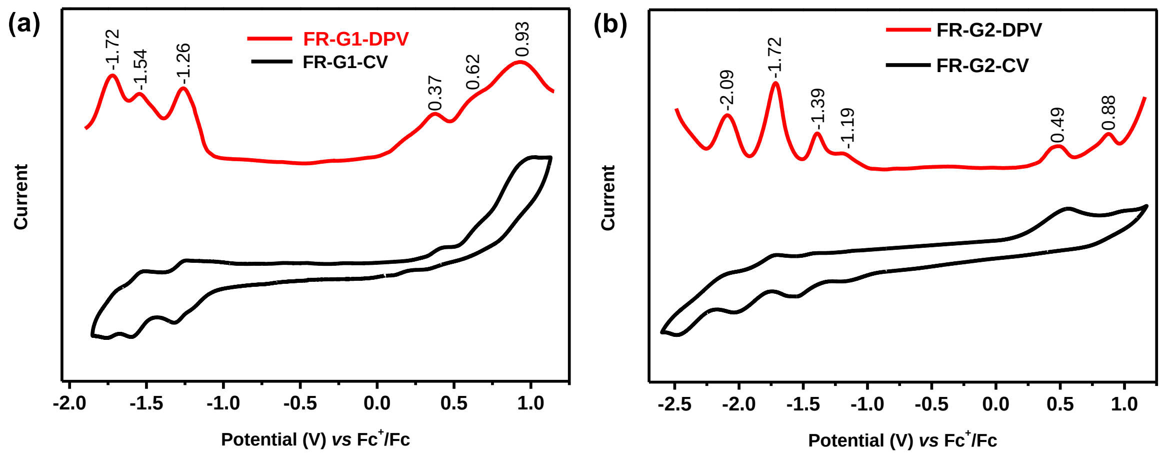

Fig. S10 Cyclic voltammograms (CV) and differential pulse voltammograms (DPV) of **FR-G1** and **FR-G2** measured in DCM.

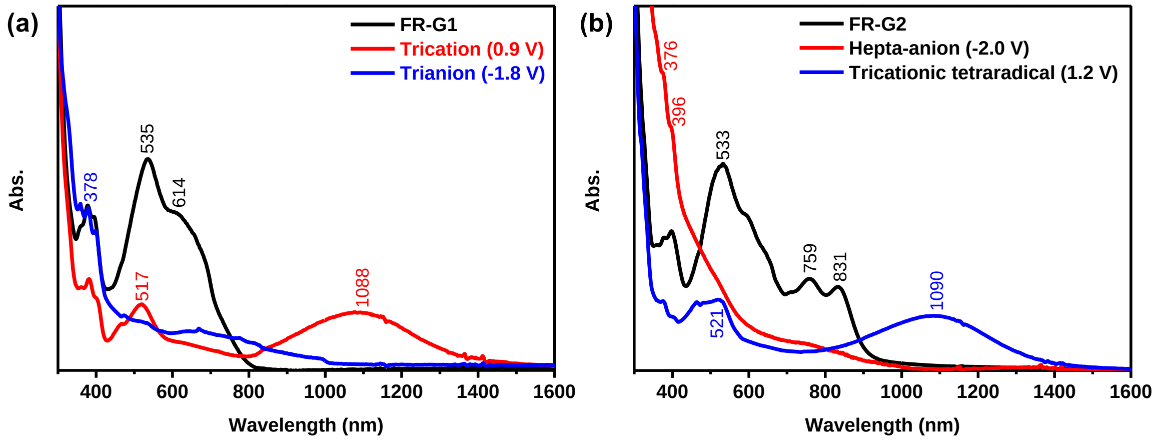

Fig. S11 UV-vis-NIR spectra of neutral, oxidized and reduced species of **FR-G1** (a) and **FR-G2** (b) in DCM during the spectro-electrochemical measurements. The applied potential is *vs* $E(Fc^+/Fc)$.

3. Electronic structure calculations

Molecular geometries of **FR-G1** and **FR-G2** have been obtained at the UB3LYP/6-31G(d,p) level with Gaussian 09 package[4] utilizing a high performance computing cluster facility of NUS. The radical nature of the electronic ground state and transition energies to higher states were calculated using the restricted active space spin flip method (RAS-SF) and the 6-31G(d) basis set with Q-Chem 4.3 package.[5] RAS-SF calculations for **FR-G1** (**FR-G2**) were performed with a 5 (9) electrons in 5 (9) orbitals in the RAS2 subspace, and with all occupied and virtual orbitals within ±0.55 (±0.30) Hartrees below and above the RAS2 orbital space for the RAS1 and RAS3 subspaces, respectively. RAS-SF wave functions were obtained from the ROHF quartet and octet for **FR-G1** and **FR-G2,** respectively. The radical character degree of the ground state singlet was estimated by the number of unpaired electrons (N_U) according to equation: $N_U = \sum_i (1 - abs(1 - n_i))$, where $\{n_i\}$ are the natural occupation numbers from the one-particle density matrix.[6] Spin densities were obtained at the DFT level (UB3LYP) and with the 6-31G(d) basis set.

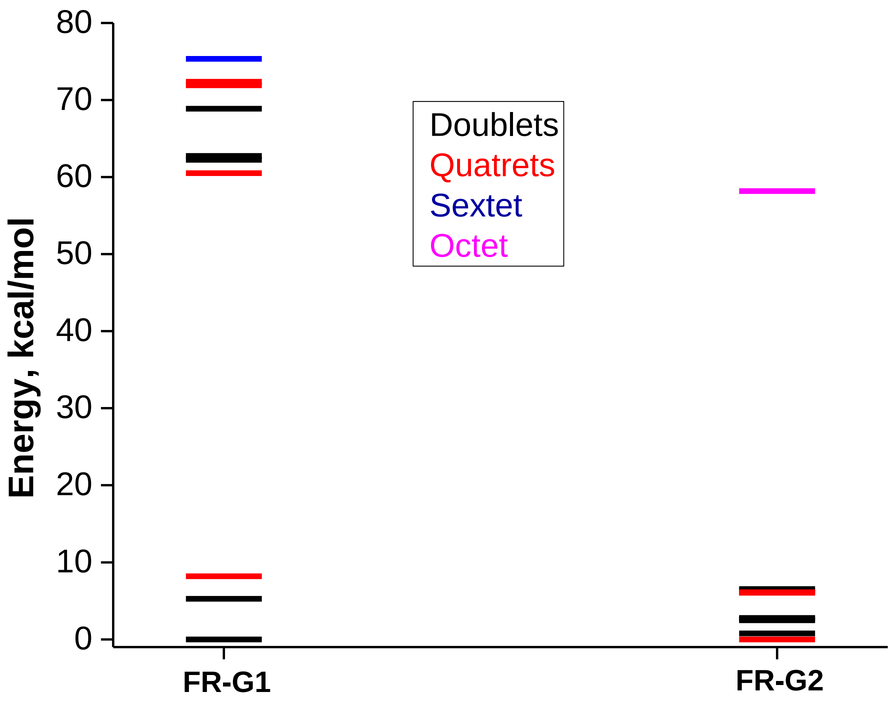

Fig. S12 Calculated (RAS-SF/6-31G*) excitation energies from the ground state and the higher energy excited states of **FR-G1** and **FR-G2**.

Table S1. Calculated relative vertical excitation energies of **FR-G1** for different spin states, and the number of unpaired electron (N_U) and electronic occupancy of SONOs at the doublet ground state.

state	multiplicity	*E*, kcal/mol	N_U	*n*(SONO-1)	*n*(SONO)	*n*(SONO+1)
D_0	doublet	0.00	1.82	1.63	1.00	0.37
D_1	doublet	5.26	2.08	1.48	1.00	0.51
D_2	doublet	62.25	2.82	1.32	0.98	0.69
D_3	doublet	62.75	3.67	1.18	0.98	0.82
D_4	doublet	68.87	3.22	1.26	1.17	0.82
Q_1	quartet	8.19	3.08	1.00	1.00	1.00
Q_2	quartet	60.52	3.66	1.04	1.00	0.97
Q_3	quartet	71.91	4.56	1.01	0.99	0.95
Q_4	quartet	72.37	4.21	1.16	1.00	0.83
S_1	sextet	75.35	5.00	1.00	1.00	1.00

D_0 state mainly corresponds to configuration indicated in the molecular orbital diagram (Fig. 2a). D_1 state corresponds to single electron excitations from D_0 within the SOMO-1, SOMO and SOMO+1 space. Q_1 state corresponds to the single electron occupation of SOMO-1, SOMO and SOMO+1 space. Higher doublet and quartet states involve excitations from the $HOMO_A$ (anthracene) and/or to the $LUMO_A$ (anthracene).

Table S2. Calculated relative vertical excitation energies of **FR-G2** for different spin states.

state	multiplicity	*E*, kcal/mol
Q_0	quartet	0.00
Q_1	quartet	6.04
Q_2	quartet	6.09
Q_3	quartet	6.11
D_1	doublet	0.78
D_2	doublet	2.47
D_3	doublet	2.65
D_4	doublet	2.77
D_5	doublet	6.32
D_6	doublet	6.53
O_1	octet	58.18

Q_0 mainly corresponds to the single electron occupation of SOMO-1, SOMO and SOMO+1. D_1 mainly corresponds to the single electron occupation of SOMO-1, SOMO and SOMO+1 (like Q_0). D_2, D_3 and D_4 are obtained as single electron excitations from D_1 state involving orbitals from SOMO-2 to SOMO+2. Q_1, Q_2 and Q_3 are obtained as single electron excitations from D_1 state involving orbitals from SOMO-2 to SOMO+2. D_5, D_6 are single excitations from D_2, D_3 and D_4 configurations involving the entire set of SOMOs.

Table S3. Calculated number of unpaired electron (N_U) and electronic occupancy of SONOs at the quartet ground state of **FR-G2**.

state	N_U	n_{SONO-3}	n_{SONO-2}	n_{SONO-1}	n_{SONO}	n_{SONO+1}	n_{SONO+2}	n_{SONO+3}
Q_0	5.23	1.50	1.42	1.00	1.00	1.00	0.58	0.50
Q_1	5.50	1.43	1.24	1.10	1.00	0.89	0.76	0.57
Q_2	5.68	1.37	1.22	1.10	1.00	0.90	0.78	0.63

Q_3	5.59	1.41	1.26	1.06	1.00	0.93	0.74	0.59
D_1	4.71	1.64	1.42	1.12	1.00	0.88	0.58	0.36
D_2	4.32	1.62	1.54	1.22	1.00	0.78	0.46	0.38
D_3	4.38	1.60	1.52	1.22	1.00	0.78	0.48	0.40
D_4	4.90	1.47	1.37	1.24	1.00	0.75	0.63	0.53
D_5	5.08	1.61	1.37	1.01	0.99	0.99	0.63	0.39
D_6	5.56	1.43	1.22	1.10	1.00	0.90	0.78	0.56
O_1	9.00	1.00	1.00	1.00	1.00	1.00	1.00	1.00

Table S4. Equilibrium enthalpy (kcal/mol), entropy (cal/mol), and Gibbs (kcal/mol) free (kcal/mol) energy differences between double and quartet states (D-Q) of **FR-G2** computed at B3LYP/6-31G* level. All values have been calculated at 298.15 K. ΔG value confirms the quartet state multiplicity of the ground state.

ΔH	ΔS	ΔG
2.26	2.24	1.59

Simulation of the absorption spectra

TDDFT calculations of the lowest electronic excitations of **FR-G1** (Table S5 and Figure S13) indicate that the electronic transitions contributing to the long-wave region of the spectrum (experimental band with a maximum at 614 nm) correspond to delocalized π-π electronic promotions with some charge transfer character from the HOMO of the anthracene moiety to the three coupled fluorenyl units (states 1 and 2). The main excitation responsible for the band located between 500-600 nm, related to the 535 nm peak in the experimental spectrum (Figure 4), corresponds to a delocalized excitation from a doubly occupied π-orbital of fluorenyl (state 3). States 4 and 5 correspond to local excitations of the anthracene fragment, while different local π-π excitations to the fluorenyl LUMOs appear with slightly higher energies (states 6 and 7).

Simulation of the **FR-G2**'s spectrum was done for transitions up to 600 nm (Table S6 and Figure S14). These states (states 1-7) exhibit similar electronic character than the two lowest optical transition in **FR-G1**, i.e. π-π excitations delocalized over the fluorenyl moieties mixed with charge transfer configurations from the anthacene's HOMO.

Table S5. Lowest optically active (oscillator strength larger than 0.1) of **FR-G1** computed at B3LYP/6-31G* level. Excitation energies are given in nm.

state index	exc. energy	strength	main contributions
1	867.38	0.4582	SOMO-1 → SOMO β
2	728.33	0.2501	SOMO-1 → SOMO+1 β
			SOMO-1 → SOMO+1 α
3	542.86	0.1725	HOMO-3 → SOMO β
4	395.89	0.2520	SOMO → LUMO+5 α
			SOMO → LUMO+4 α
			SOMO-1 → LUMO+1 α
5	391.56	0.1681	HOMO → LUMO β
			HOMO → LUMO α
6	358.63	0.1150	HOMO-1 → LUMO+4 β
			HOMO-1 → LUMO+5 β
7	357.53	0.1030	HOMO-3 → LUMO+1 β

Table S6. Lowest optically active (oscillator strength larger than 0.1) of **FR-G2** computed at B3LYP/6-31G* level. Excitation energies are given in nm.

state index	exc. energy	strength	main contributions
1	1111.22	0.1086	SOMO-2 → SOMO-1 β
2	1039.87	0.5770	SOMO-1 → SOMO+2 α
3	868.82	0.2772	SOMO-3 → SOMO+2 α
			SOMO-2 → SOMO+3 β
4	858.80	0.1889	SOMO-2 → SOMO β
			SOMO-2 → SOMO+1 β
5	848.94	0.5169	SOMO-2 → SOMO+1 β
			SOMO-2 → SOMO β
6	840.52	0.1531	HOMO → SOMO-1 β
			SOMO-1 → SOMO+3 α
7	722.04	0.3565	SOMO-2 → SOMO+2 β
			SOMO-2 → SOMO+2 α

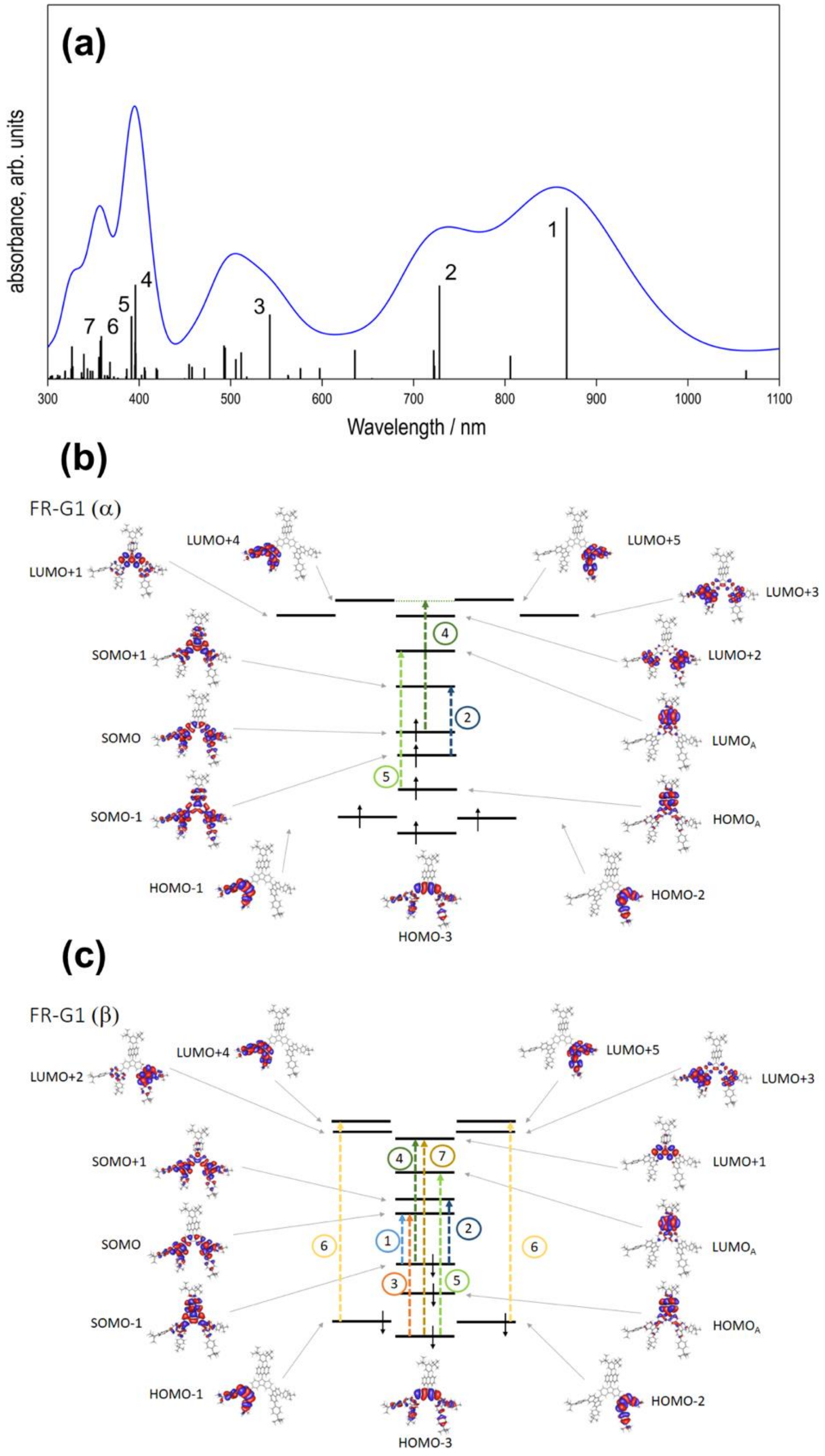

Fig. S13 Simulated absorption spectra of **FR-G1** (a) and the main spin-α (b) and spin-β (c) orbital-to-orbital contributions to the lowest optically active transitions computed at the B3LYP/6-31G* level.

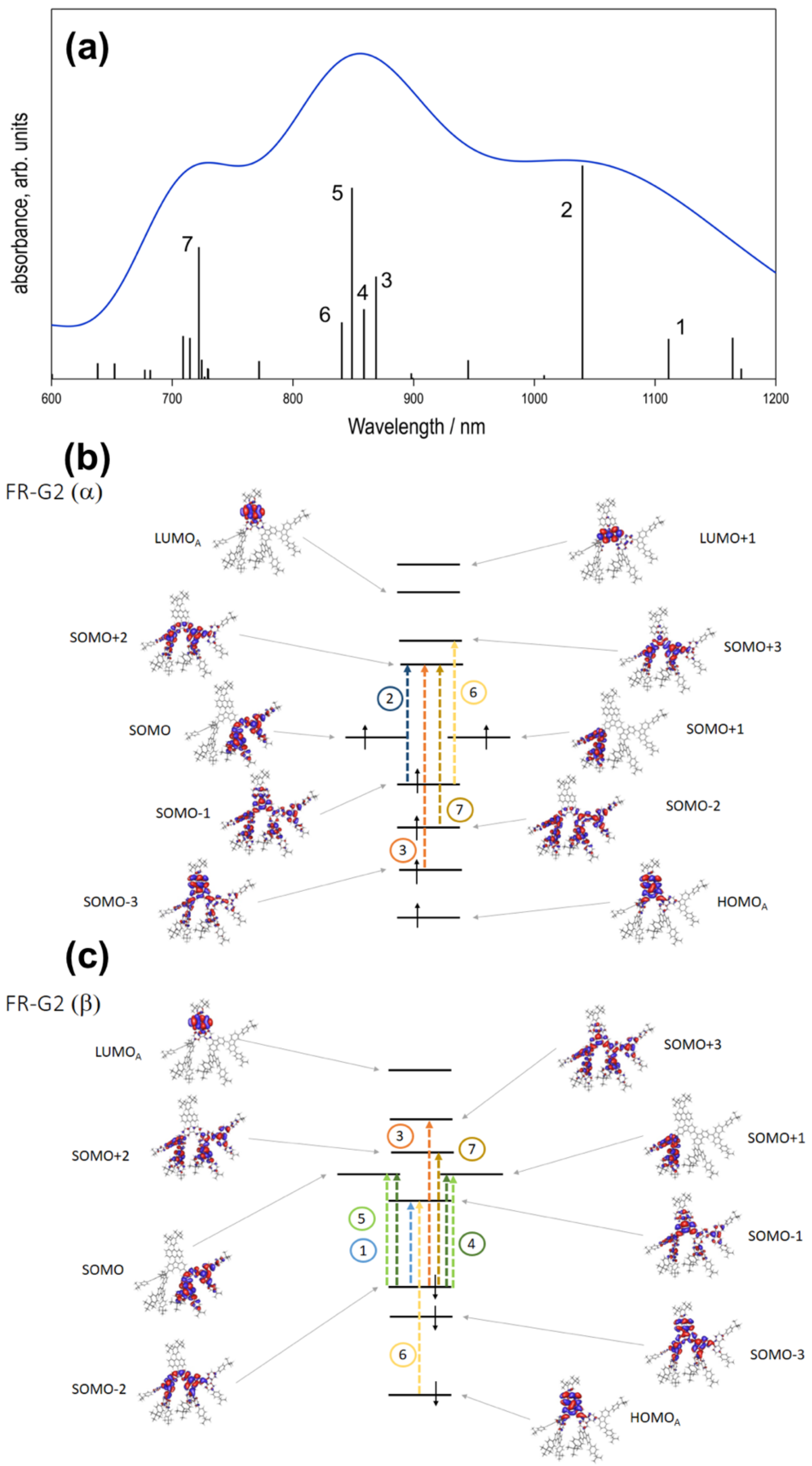

Fig. S14 Simulated absorption spectra of **FR-G2** (a) and the main spin-α (b) and spin-β (c) orbital-to-orbital contributions to the lowest optically active transitions computed at the B3LYP/6-31G* level.

4. Additional NMR and mass spectra

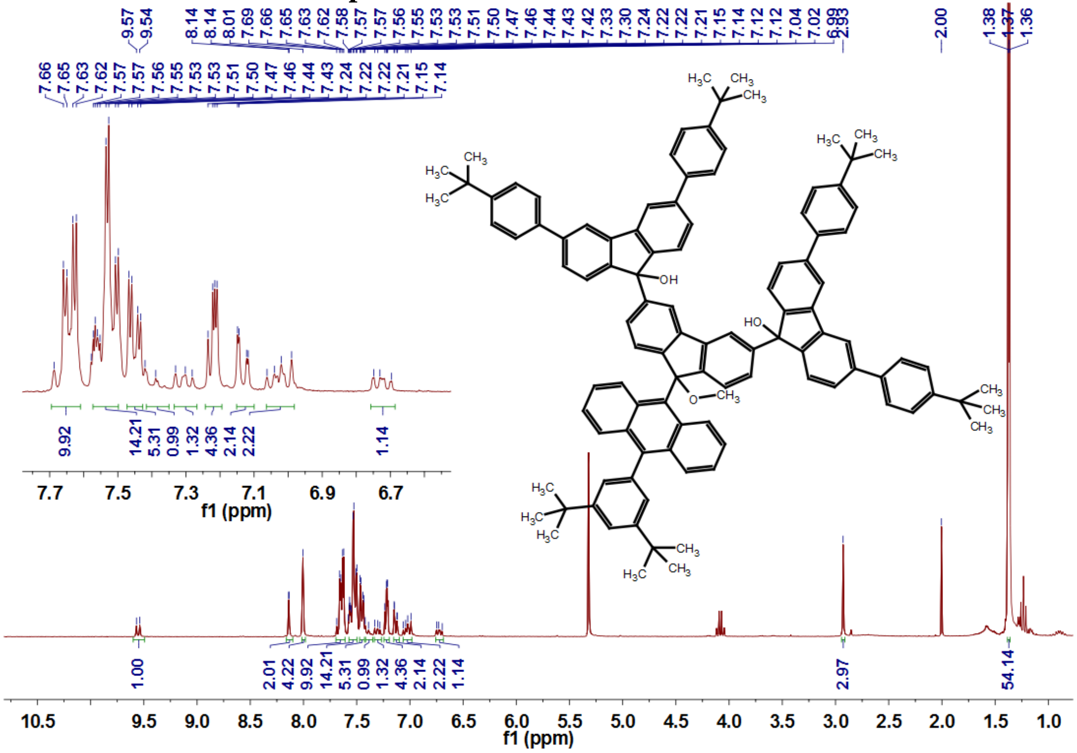

Fig. S15 ^{1}H NMR spectrum of **2** (300 MHz, CD_2Cl_2, rt, some residual EA)

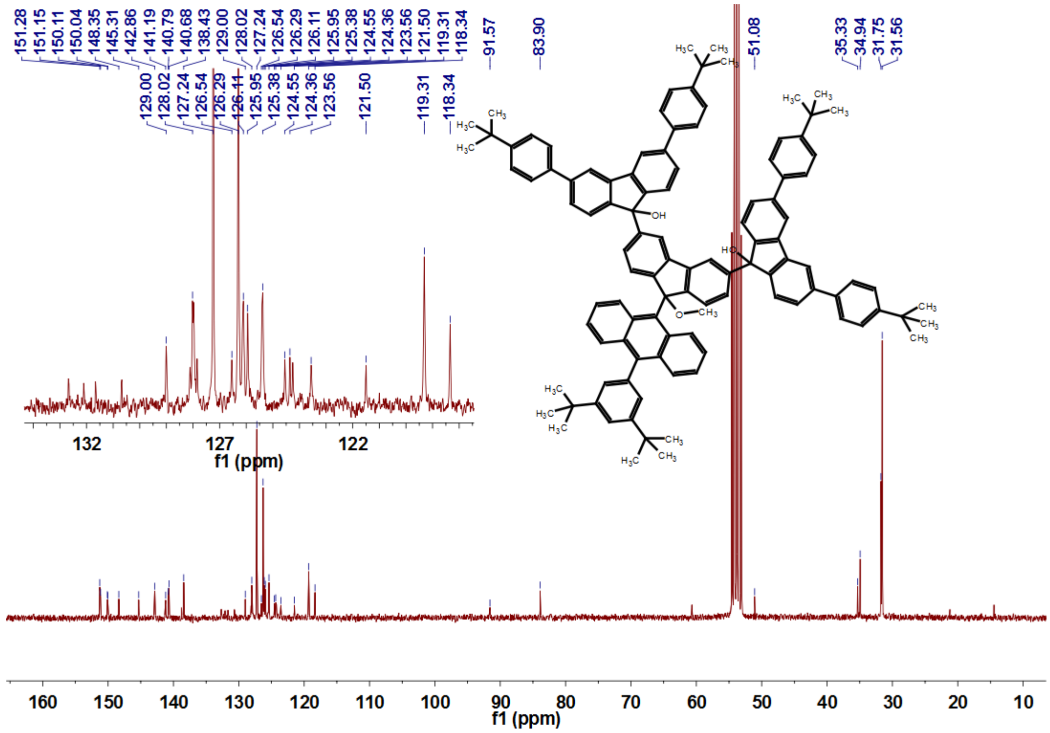

Fig. S16 ^{13}C NMR spectrum of **2** (75 MHz, CD_2Cl_2, rt, some residual EA)

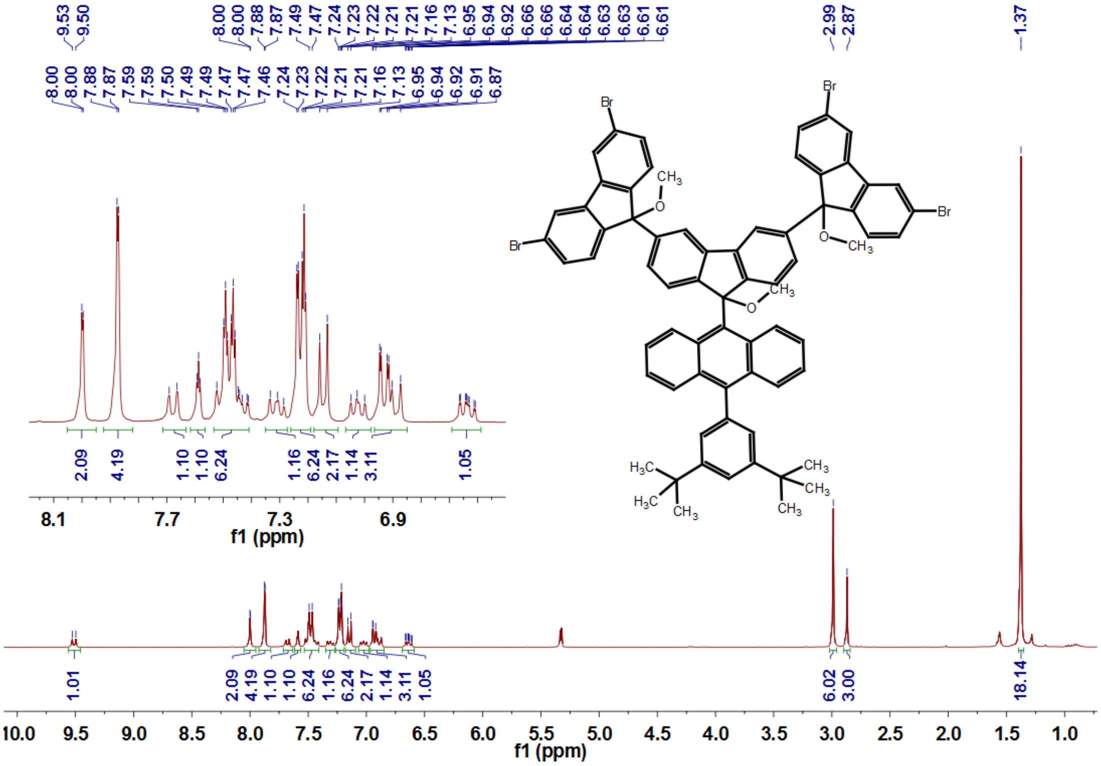

Fig. S17 ^{1}H NMR spectrum of compound **3** (300 MHz, CD_2Cl_2, rt)

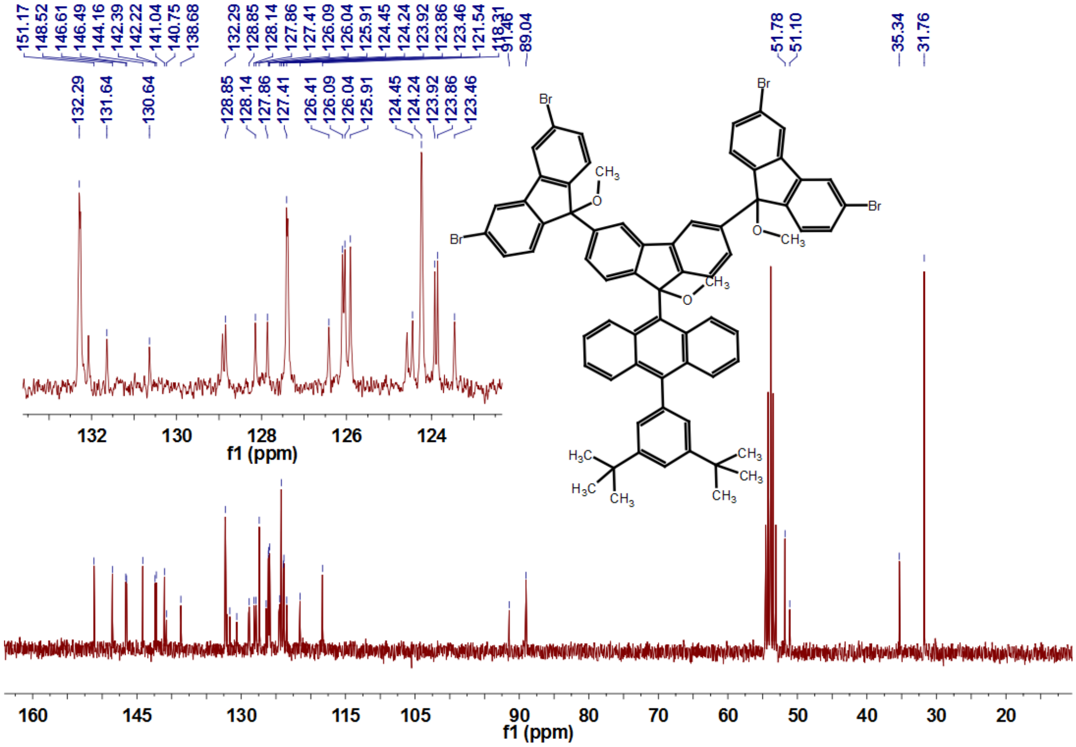

Fig. S18 ^{13}C NMR spectrum of compound **3** (75 MHz, CD_2Cl_2, rt)

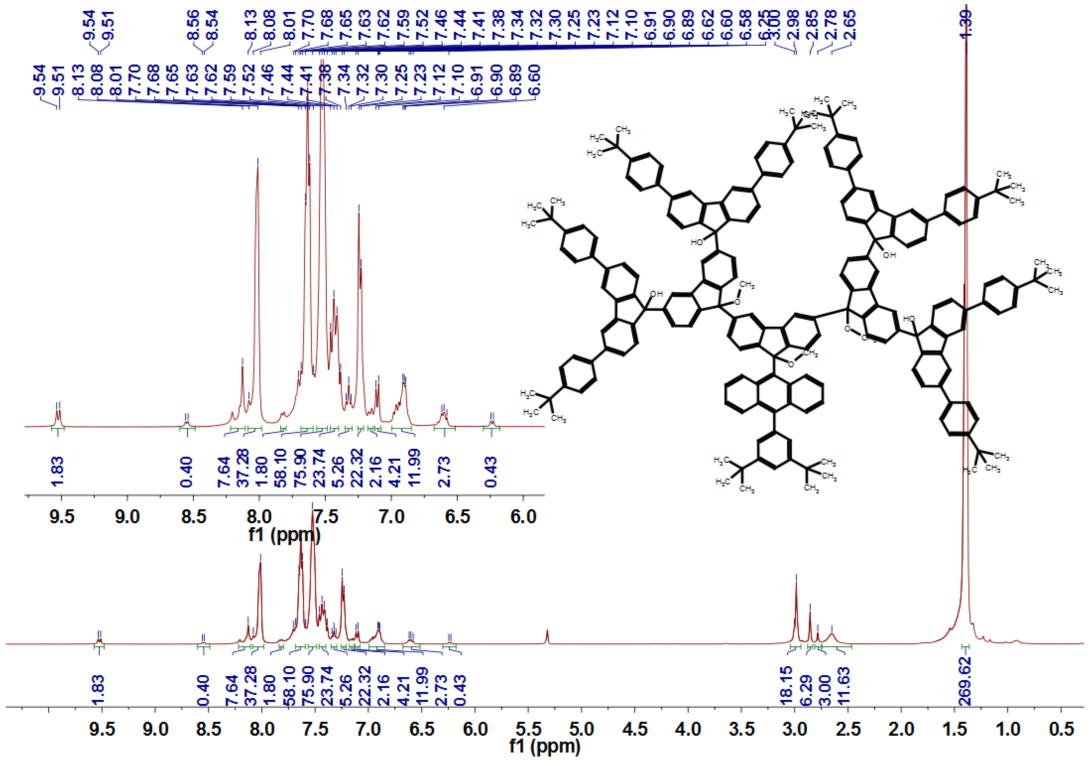

Fig. S19 ^{1}H NMR spectrum of compound **4** (400 MHz, CD_2Cl_2, rt)

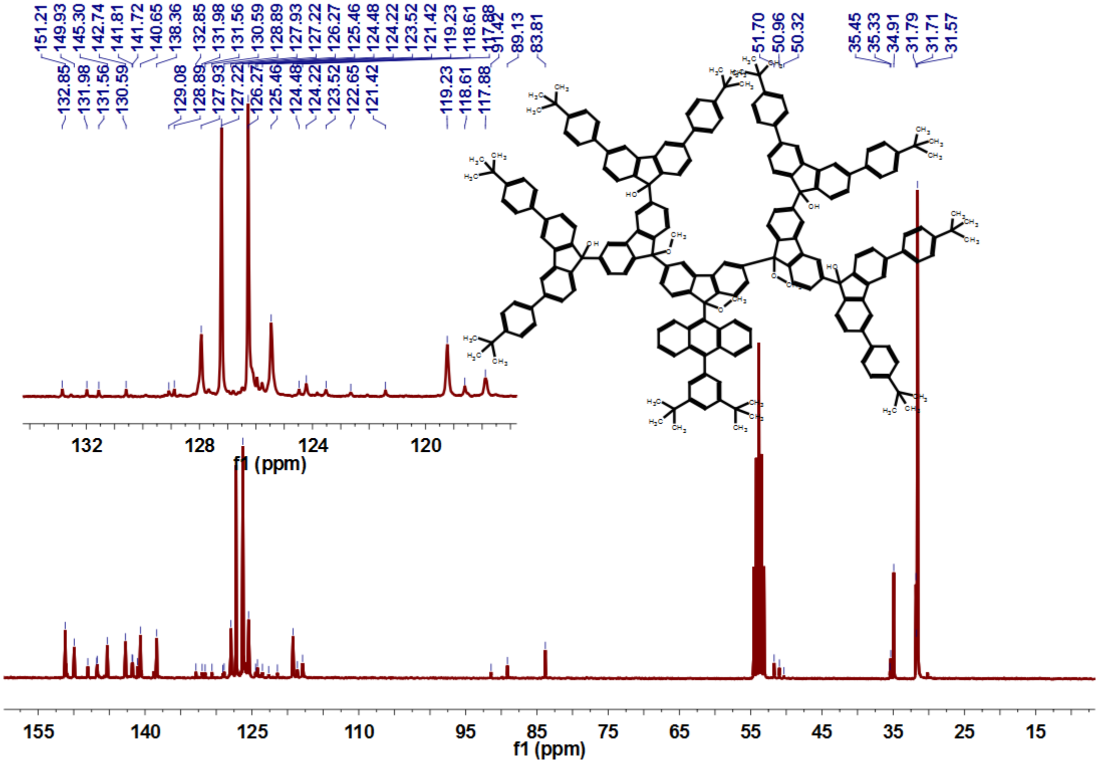

Fig. S20 ^{13}C NMR spectrum of compound **4** (75 MHz, CD_2Cl_2, rt)

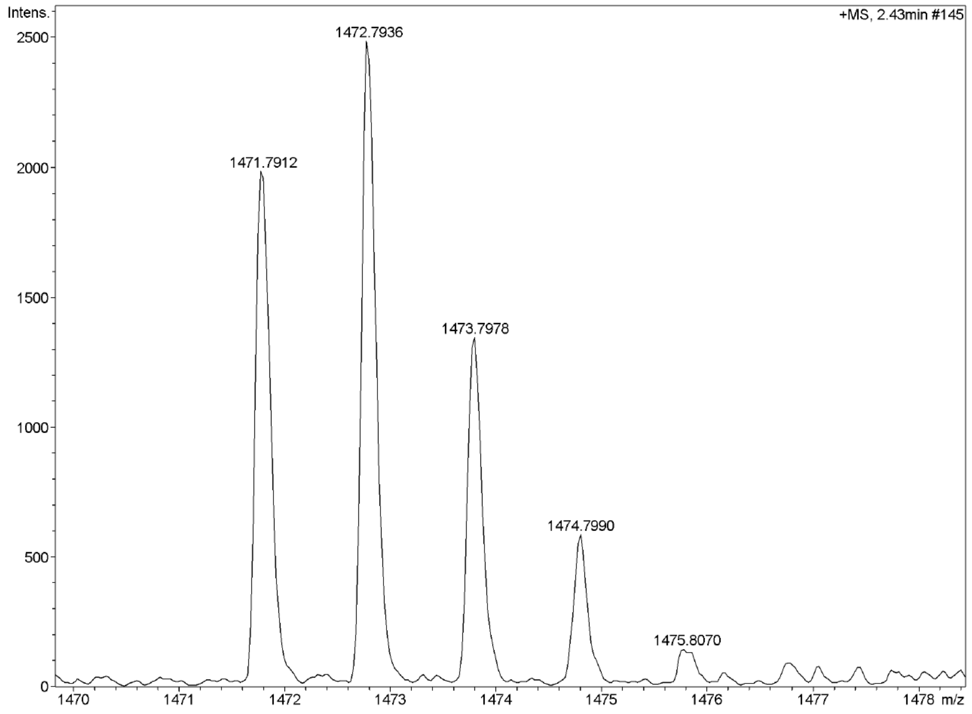

Fig. S21 HR mass spectrum (ESI) of **2**.

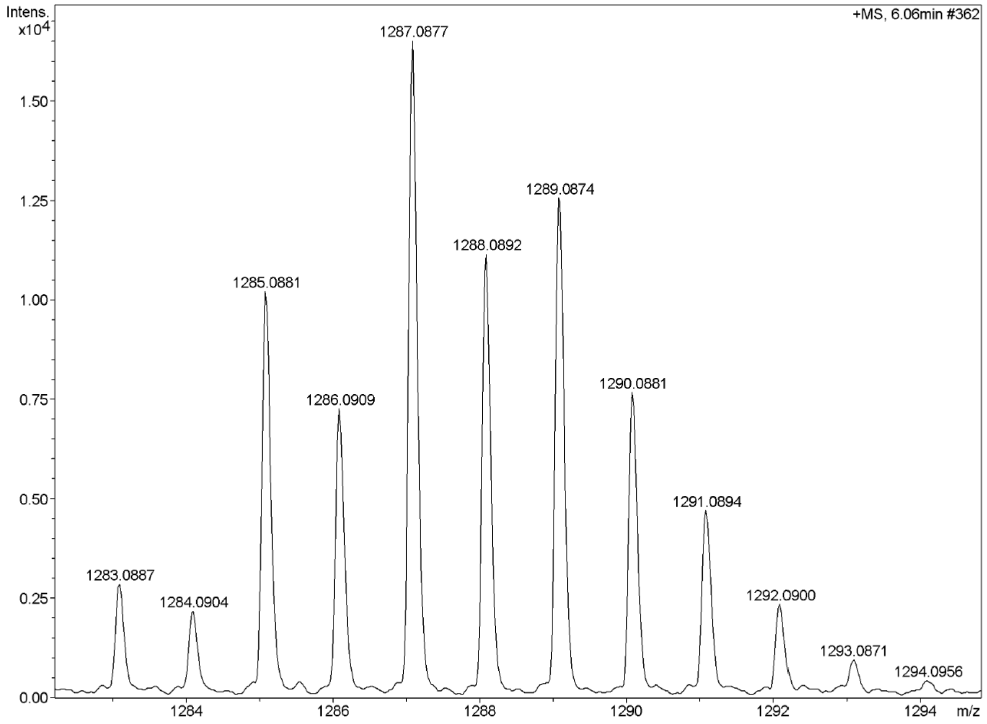

Fig. S22 HR mass spectrum (ESI) of **3**.

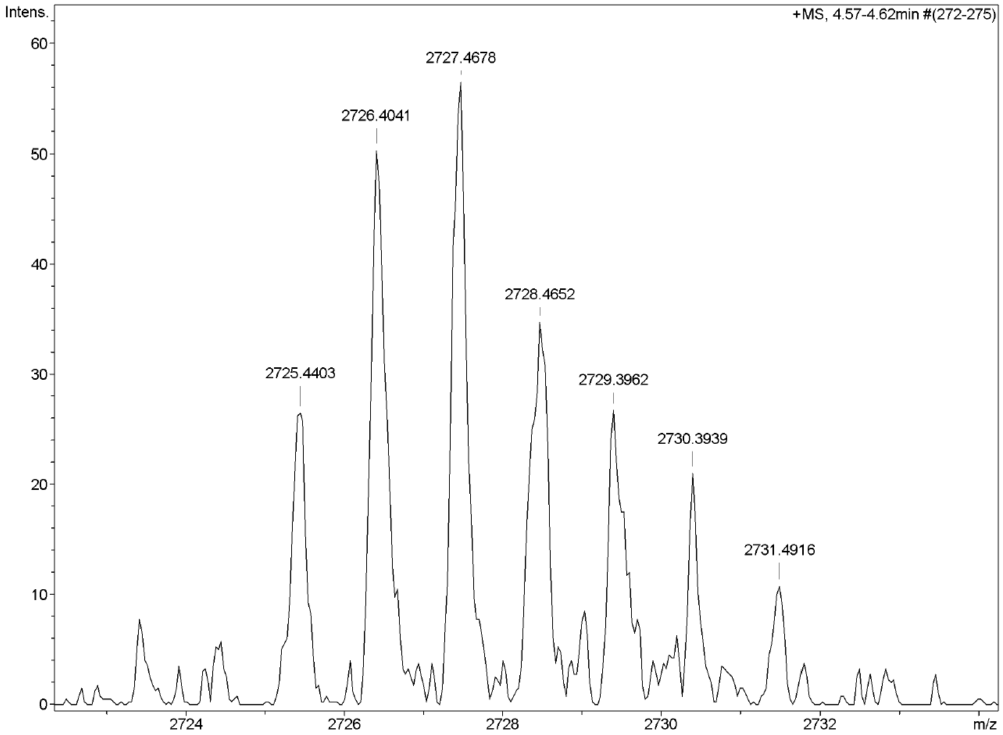

Fig. S23 HR mass spectrum (APCI) of **4**.

5. References

1. X. Lu, S. Lee, J. O. Kim, T. Y. Gopalakrishna, H. Phan, T. S. Herng, Z. Lim, Z. Zeng, J. Ding, D. Kim and J. Wu, *J. Am. Chem. Soc.*, 2016, **138**, 13048.

2. H.-C. Ting, C.-H. Tsai, J.-H. Chen, L.-Y. Lin, S.-H. Chou, K.-T. Wong, T.-W. Huang and C.-C. Wu, *Org. Lett.*, 2012, **14**, 6338.

3. J. H. Van Vleck, "The theory of electric and magnetic susceptibilities", Oxford University Press, Oxford, **1932**.

4. *Gaussian 09; Revision A.2*; Frisch, M. J.; Trucks, G. W.; Schlegel, H. B.; Scuseria, G. E.; Robb, M. A.; Cheeseman, J. R.; Scalmani, G.; Barone, V.; Mennucci, B.; Petersson, G. A.; Nakatsuji, H.; Caricato, M.; Li, X.; Hratchian, H. P.; Izmaylov, A. F.; Bloino, J.; Zheng, G.; Sonnenberg, J. L.; Hada, M.; Ehara, M.; Toyota, K.; Fukuda, R.; Hasegawa, J.; Ishida, M.; Nakajima, T.; Honda, Y.; Kitao, O.; Nakai, H.; Vreven, T.; Montgomery, J., J. A.; Peralta, J. E.; Ogliaro, F.; Bearpark, M.; Heyd, J. J.; Brothers, E.; Kudin, K. N.; Staroverov, V. N.; Kobayashi, R.; Normand, J.; Raghavachari, K.; Rendell, A.; Burant, J. C.; Iyengar, S. S.; Tomasi, J.; Cossi, M.; Rega, N.; Millam, N. J.; Klene, M.; Knox, J. E.; Cross, J. B.; Bakken, V.; Adamo, C.; Jaramillo, J.; Gomperts,

R.; Stratmann, R. E.; Yazyev, O.; Austin, A. J.; Cammi, R.; Pomelli, C.; Ochterski, J. W.; Martin, R. L.; Morokuma, K.; Zakrzewski, V. G.; Voth, G. A.; Salvador, P.; Dannenberg, J. J.; Dapprich, S.; Daniels, A. D.; Farkas, Ö.; Foresman, J. B.; Ortiz, J. V.; Cioslowski, J.; Fox, D. J.; Gaussian, Inc., Wallingford CT, **2009**.

5. Shao, Y.; Gan, Z.; Epifanovsky, E.; Gilbert, A.T.B.; Wormit, M.; Kussmann, J.; Lange, A.W.; Behn, A.; Deng, J.; Feng, X.; Ghosh, D.; Goldey, M.; Horn, P.R.; Jacobson, L.D.; Kaliman, I.; Khaliullin, R.Z.; Kus, T.; Landau, A.; Liu, J.; Proynov, E.I.; Rhee, Y.M.; Richard, R.M.; Rohrdanz, M.A.; Steele, R.P.; Sundstrom, E.J.; Woodcock III, H.L.; Zimmerman, P.M.; Zuev, D.; Albrecht, B.; Alguires, E.; Austin, B.; Beran, G.J.O.; Bernard, Y.A.; Berquist, E.; Brandhorst, K.; Bravaya, K.B.; Brown, S.T.; Casanova, D.; Chang, C.-M.; Chen, Y.; Chien, S.H.; Closser, K.D.; Crittenden, D.L.; Diedenhofen, M.; DiStasio Jr., R.A.; Do, H.; Dutoi, A.D.; Edgar, R.G.; Fatehi, S.; Fusti-Molnar, L.; Ghysels, A.; Golubeva-Zadorozhnaya, A.; Gomes, J.; Hanson-Heine, M.W.D.; Harbach, P.H.P.; Hauser, A.W.; Hohenstein, E.G.; Holden, Z.C.; Jagau, T.-C.; Ji, H.; Kaduk, B.; Khistyaev, K.; Kim, J.; Kim, J.; King, R.A.; Klunzinger, P.; Kosenkov, D.; Kowalczyk, T.; Krauter, C.M.; Laog, K.U.; Laurent, A.; Lawler, K.V.; Levchenko, S.V.; Lin, C.Y.; Liu, F.; Livshits, E.; Lochan, R.C.; Luenser, A.; Manohar, P.; Manzer, S.F.; Mao, S.-P.; Mardirossian, N.; Marenich, A.V.; Maurer, S.A.; Mayhall, N.J.; Oana, C.M.; Olivares-Amaya, R.; O'Neill, D.P.; Parkhill, J.A.; Perrine, T.M.; Peverati, R.; Pieniazek, P.A.; Prociuk, A.; Rehn, D.R.; Rosta, E.; Russ, N.J.; Sergueev, N.; Sharada, S.M.; Sharmaa, S.; Small, D.W.; Sodt, A.; Stein, T.; Stuck, D.; Su, Y.-C.; Thom, A.J.W.; Tsuchimochi, T.; Vogt, L.; Vydrov, O.; Wang, T.; Watson, M.A.; Wenzel, J.; White, A.; Williams, C.F.; Vanovschi, V.; Yeganeh, S.; Yost, S.R.; You, Z.-Q.; Zhang, I.Y.; Zhang, X.; Zhou, Y.; Brooks, B.R.; Chan, G.K.L.; Chipman, D.M.; Cramer, C.J.; Goddard III, W.A.; Gordon, M.S.; Hehre, W.J.; Klamt, A.; Schaefer III, H.F.; Schmidt, M.W.; Sherrill, C.D.; Truhlar, D.G.; Warshel, A.; Xu, X.; Aspuru-Guzik, A.; Baer, R.; Bell, A.T.; Besley, N.A.; Chai, J.-D.; Dreuw, A.; Dunietz, B.D.; Furlani, T.R.; Gwaltney, S.R.; Hsu, C.-P.; Jung, Y.; Kong, J.; Lambrecht, D.S.; Liang, W.Z.; Ochsenfeld, C.; Rassolov, V.A.; Slipchenko, L.V.; Subotnik, J.E.; Van Voorhis, T.; Herbert, J.M.; Krylov, A.I.; Gill, P.M.W.; Head-Gordon, M. *Mol. Phys.*, **2015**, *113*, 184.

6. (a) M. Head-Gordon, *Chem. Phys. Lett.*, 2003, **372**, 508; (b) D. Casanova and M. Head-Gordon, *Phys. Chem. Chem. Phys.*, 2009, **11**, 9779.
